# Validity and Acceptability of Kimberley Mum’s Mood Scale to Screen for Perinatal Anxiety and Depression in Remote Aboriginal Health Care Settings

**DOI:** 10.1371/journal.pone.0168969

**Published:** 2017-01-30

**Authors:** Julia V. Marley, Jayne Kotz, Catherine Engelke, Melissa Williams, Donna Stephen, Sudha Coutinho, Stephanie K. Trust

**Affiliations:** 1 The Rural Clinical School of Western Australia, The University of Western Australia, Broome, Western Australia, Australia; 2 Kimberley Aboriginal Medical Services Ltd, Broome, Western Australia, Australia; 3 School of Psychology and Exercise Science, Murdoch University, Murdoch, Western Australia, Australia; 4 The Rural Clinical School of Western Australia, The University of Western Australia, Kununurra, Western Australia, Australia; 5 Western Australian County Health Service, Broome, Western Australia, Australia; 6 Independent Consultant, Broome, Western Australia, Australia; 7 Kununurra Medical, Kununurra, Western Australia, Australia; Royal Children's Hospital, AUSTRALIA

## Abstract

**Background:**

The Edinburgh Postnatal Depression Scale (EPDS) is widely recommended for perinatal anxiety and depression screening. However, many Aboriginal women find EPDS language complex and confusing, and providers find using it with Aboriginal women challenging. The two part Kimberley Mum’s Mood Scale (KMMS) was developed to improve screening: Part 1 is a Kimberley version of EPDS; Part 2 is a psychosocial tool that enables contextualisation of Part 1 scores. We aimed to determine if KMMS is a valid and acceptable method of identifying Kimberley Aboriginal perinatal women at risk of anxiety or depressive disorders compared to a semi-structured clinical interview.

**Methods:**

Across 15 sites in the Kimberley, Western Australia, 97 Aboriginal women aged 16 years and older who intended to continue with their pregnancy or had a baby within the previous 12 months were administered the KMMS by trained healthcare providers who provided an overall assessment of no, low, moderate or high risk; 91 participants were then independently assessed by a blinded clinical expert using Diagnostic and Statistical Manual of Mental Disorders, 4th Edition criteria. A qualitative approach was used to determine KMMS’ acceptability.

**Results:**

Part 1 had high internal consistency (Cronbach’s alpha, 0.89), and overall KMMS risk equivalence for screening for anxiety or depressive disorders was moderate (sensitivity, 83%; specificity, 87%; positive predictive value, 68%). Participants found the process easy and useful, and healthcare providers found KMMS more useful than EPDS. Part 2 allowed healthcare providers to ask questions that gave participants an opportunity to express themselves, resulting in a deeper understanding between them.

**Conclusion:**

KMMS is an effective tool for identifying Kimberley Aboriginal perinatal women at risk of anxiety and depressive disorders. Adoption of KMMS with culturally safe training and support is likely to improve screening processes, and with further validation may have broader applicability across remote Australia.

## Introduction

Mental health during and after pregnancy is important for the well-being of mother and infant. Unfortunately anxiety and depressive disorders affect 20% of Australian pregnant women and mothers within the first year post birth [1, 2]. Potential long-term health impacts include poorer birth outcomes [3–6], poorer bonding between mother and infant [7, 8], and susceptibility to lifelong psychopathology [9, 10]. Early identification and appropriate support may improve outcomes, however most women do not seek and/or receive treatment [11, 12].

Mental health prior to pregnancy is an important predictor of perinatal wellbeing. A recent 20 year prospective Australian cohort study (382 women with 560 pregnancies) found that 85% of women with perinatal depression had mental health issues prior to pregnancy [13]. Among Aboriginal and Torres Strait Islander peoples loss and grief is pervasive with high levels of post-traumatic stress and trans-generational trauma [14]. Few access mental health support early [15], hence screening and timely culturally appropriate early intervention for perinatal anxiety and depression could result in significant benefits.

Screening processes need to be acceptable to patients and staff, and seen to be easy to use and useful, or they are unlikely to be well implemented. The Edinburgh Postnatal Depression Scale (EPDS) is a widely recommended screening tool for perinatal depression and anxiety in Australia and internationally [16–18]. In the Kimberley region of north Western Australia its use has been in regional guidelines since 2010. However, many Aboriginal women find the EPDS language complex and confusing, providers find using it with Aboriginal women challenging, and hence uptake has been limited amongst Aboriginal women [19].

Linguistic, cultural and practical reasons highlight the need for an alternative approach to screening in remote Aboriginal populations. Few mental health screening tools have been developed and validated against a reference standard for use among Aboriginal and Torres Strait Islander populations [18, 20, 21]. The EPDS has been translated for Mount Isa and Townsville Aboriginal populations [22]. While these tools may be linguistically appropriate, they have not been validated against a reference standard assessment. Community consultation with Kimberley Aboriginal women and key collaborators also demonstrated the need for a culturally safe screening tool that included adequate ‘yarning time’ [23]. This led to the development of the Kimberley Mum’s Mood Scale (KMMS). We aimed to determine if the KMMS is a reliable, valid and acceptable tool for identifying Kimberley Aboriginal women at risk of perinatal anxiety or depressive disorders when compared to a diagnosis from a blinded clinical expert.

## Methods

Data collection was between 9 May 2013 and 11 June 2014. At 15 Kimberley sites Aboriginal women aged 16 years and older, with on-going pregnancy or birth within the previous 12 months were invited to participate. Women were excluded if they were: unable to provide informed consent; less than 7 days post-partum; less than 6 weeks pregnant; known to be currently acutely mentally unwell; or active clients of the Department for Child Protection and Family Support. Recruitment was opportunistic through maternal and child health clinics and snowballing recruitment through family and community.

A cross-sectional design was used to determine the reliability and validity of the KMMS to identify women with anxiety and/or depressive disorders compared to a reference standard. A qualitative approach was used to determine KMMS’ acceptability.

### KMMS

The KMMS was originally developed by Kimberley health care providers in collaboration with over 100 Aboriginal women from eight Kimberley language groups [23]. An iterative development of wording of the 10 questions used in the EDPS began using the Mt Isa and Townsville translated versions [22]. Multiple focus groups provided input for words, visuals, and a protocol for administration. They also highlighted the need for Part 2 that includes the time and opportunity to talk about issues that are important to them [23]. The two part KMMS is designed for administration by midwives, child health nurses (CHNs) and other providers of perinatal care [23, 24].

Part 1 covers the same areas as the EPDS using similar stems and scoring system (0, 1, 2, or 3 according to the severity of the symptom, total score up to 30 for the 10 questions) [23]. As a result of this process the 10 questions that comprise Part 1 uses ‘Kimberley’ English, locally developed graphics and a visual Likert scale focusing on feelings not numbers. This visual scale represents women’s faces from happy through neutral to sad with overhead sun and clouds to match mood, so sun is happy and clouds darken with mood (Figure A in S1 File).

Part 2 complements information gathered in Part 1 by providing a more comprehensive understanding of a woman’s story. It is a psychosocial tool, based on the SAFE START Guidelines [25], that incorporates key issues identified by Kimberley Aboriginal women: stressors, self-esteem, relationships, childhood experiences and mental health including substance use. A Part 2 psychosocial screening guideline was produced to guide questions for health care providers relating to these six key domains for anxiety and depression (Figure A in S1 File). Discussion of protective and risk factors around these domains enables the health care provider administering the KMMS to put the score from Part 1 into context and identify the factors that influences a woman’s social and emotional wellbeing. The clinical decision regarding the level of risk of depression and/or anxiety is moderated by protective factors and increased by factors such as the level of acute psychiatric distress or crisis, current abuse (including family violence), current drug and alcohol abuse and the presence of multiple environmental risk factors.

A key component of Part 2’s assessment is provision of a mental health brief intervention, which can include giving basic information about perinatal mental health; assisting the woman to develop action plans to identify problems, goals, strategies, support and arranging follow-up; as well as referral to a general practitioner (GP) or other supportive services as required. Health care providers can also use this opportunity to provide intensive support, which has been shown to be useful in preventing postnatal depression in the general population [26, 27] and to develop an appropriate management plan.

### Training study personnel

Midwives and/or CHNs volunteered to be study personnel and received 4–6 hours of training. Trainers included a senior mental health clinician (SC) and two experienced midwives/CHNs (MW, JK). Training encompassed: current context of perinatal mental health screening; dealing with disclosure; assessing degree of risk; developing management plans; utilising local referral pathways; and the research process. Refresher training was provided as required.

### Administering the KMMS

Study personnel (n = 15), including two members of the research team (MW, DS), consented participants and administered the KMMS (Figure A in S1 File). Overall KMMS risk (no, low, moderate, high) used the score from Part 1 as a guide with discussion during Part 2 (assessing protective and risk factors) moderating the final assessment. Participants who answered “yes always” or “yes sometimes” to Q10 (harm to self or others) were directly referred to the participant’s nominated GP or mental health professional. Debriefing interviews/counselling with the study coordinator (JK) was undertaken with study personnel. The research team facilitated study personnel administering the KMMS (30–60 minutes) in a culturally safe space, and organised appointments for the reference standard GP assessment.

### Reference standard GP assessment

The reference standard GP assessment (Figure B in S1 File) was developed by a female Kimberley Aboriginal GP (CE) with extensive community connections and accredited training and experience in mental health assessment. Diagnosis was according to Diagnostic and Statistical Manual of Mental Disorders, 4th Edition criteria for anxiety and depressive disorders [28], and severity (low, moderate, high) based on the Australian GP Mental State Examination [29]. To assess this tool’s reliability the first six cases were presented to Kimberley based Psychiatrists and GPs experienced in Aboriginal health within three days of the assessment. The externally assessed diagnosis and severity was the same as the study GP. The reference standard GP assessment was undertaken by this GP or three other equally qualified female GPs.

Participants were assessed by a study GP as soon as possible after the KMMS was administered, generally within 24-hours (maximum 7 days). The study GP was blinded to the KMMS assessment. A follow-up management plan was negotiated between participant and study GP. This contributed to ongoing clinical management and included referral to their nominated health service provider. Those with moderate or high severity anxiety and/or depressive disorders had their care immediately handed over to their nominated GP to ensure they were not lost to follow-up.

### Statistical analysis

Analyses were performed using Stata, version 13 (StataCorp). Differences in characteristics between antenatal and postnatal participants, and between participants who were or were not diagnosed with anxiety and/or depressive disorders were compared using χ^2^ tests for categorical data and Mann–Whitney tests for continuous non-parametric data. Clinical diagnoses of anxiety and depressive disorders, and antenatal and postnatal data were combined for further analysis. Internal consistency of Part 1 was determined using Cronbach’s alpha [30]. The equivalence value for i) Part 1 alone and ii) the overall KMMS risk (Part 1 and 2 combined) for identifying participants at risk of anxiety or depressive disorders compared to the reference standard GP assessment was determined from receiver operating characteristics (ROC) curves. Sensitivity, specificity, predictive values and percentage correctly classified for identifying perinatal anxiety and depressive disorders were determined based on these cut-points. *P* < 0.05 was defined as statistically significant.

### Acceptability of KMMS

Directly following KMMS administration participants completed a short questionnaire about their experience (Figure C in S1 File). After data collection study personnel were asked to complete an anonymous online questionnaire (Figure D in S1 File) and a follow-up interview with the study coordinator (JK).

Data from the questionnaires and interviews were transcribed into Microsoft Word 2010 (Microsoft) documents. Individual documents were amalgamated into a textual database using the tabular functions of Word. The research team reviewed and conducted thematic analyses of the data. The initial focus was to identify factors that explored KMMS’ acceptability as a screening tool with segments of text coded appropriately. As review of the document continued, initial coding categories were further divided and refined and a hierarchical coding scheme was developed based on consensus. The research team included two experienced Kimberley Aboriginal GPs (CE, ST) with strong community connections and Aboriginal perspectives were privileged when consensus could not be reached. Content of coding categories was reviewed and important and recurring themes identified. Conclusions were developed and tested against data from the database and quantitative data.

### Ethics approval

Ethics approval was obtained from the Human Research Ethics Committee of The University of Western Australia, the Western Australian Aboriginal Health Ethics Committee (WAAHEC) and WACHS Research Ethics Committee. This project was supported by the Kimberley Aboriginal Health Planning Forum Research Subcommittee [31]. Written consent from participants was obtained prior to enrolling them in the study.

## Results

**Fig 1:**
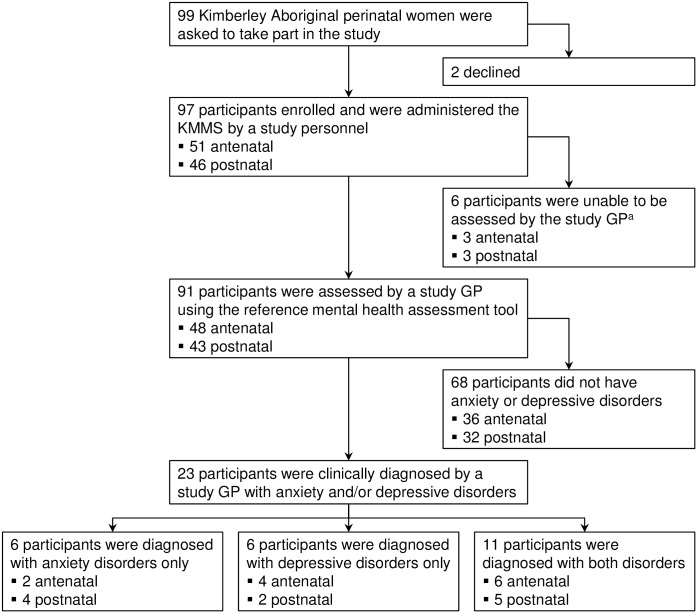
Flow of participants through the study. KMMS = Kimberley Mum’s Mood Scale. GP = general practitioner. ^a^ A study GP was unable to assess 6 participants because: i) the GP was ill at the time (n = 1); ii) participant answered “yes always” to Q10 and was immediate referred to mental health services by the study personnel administering the KMMS as high risk of self-harm (n = 1); and iii) participants did not want to wait and did not return for the reference standard GP assessment (n = 4).

Ninety nine Aboriginal women were asked to take part in the study; 97 (98%) consented and were administered the KMMS (Fig 1). All participants were to be administered both parts of the KMMS, however one study personnel did not administer Part 2 (3 participants) because she felt it was out of her scope of practice. Ninety-one of 97 (94%) participants received the reference standard GP assessment; 23 of 91 (25%) participants were clinically diagnosed with anxiety and/or depressive disorders; and 88 of 91 (97%) assessments occurred within 24-hours of the KMMS.

The only significant demographic difference between antenatal and postnatal participants was median number of children (one greater in postnatal women); and between participants who were or were not diagnosed with anxiety and/or depressive disorders was median number of people reported to be living in the house (Table 1).

**Table 1:** Characteristics of participants enrolled in the study by perinatal period and reference standard diagnosis.

Characteristic	Perinatal period		Diagnosis of anxiety and/or depressive disorders		
	Antenatal (n = 51)	Postnatal (n = 46)	No (n = 68)	Yes (n = 23)	Not done (n = 6)
Age (range)	24.3 (16.2–37.4)	24.1 (17.2–41.2)	23.6 (16.2–41.2)	25.6 (17.8–35.2)	26.4 (23.9–31.8)
Median gestational age in weeks (range)	28.5 (7–40)	-	28 (7–40)	28.5 (8–39)	35 (19–38)
Median age of baby in weeks (range)	-	20 (1–53)	17 (1–53)	25 (2–52)	24 (8–29)
Socioeconomic					
Currently employed or studying (%)	16 (31.4)	13 (28.3)	22 (32.4)	6 (26.1)	1 (16.7)
Formal education (year 10 or higher) (%)	47 (92.2)	42 (91.3)	62 (91.2)	21 (91.3)	6 (100)
Median number of children (range)	1 (0–7)	2 (1–7)*	1 (0–7)	2 (0–6)	3 (2–4)**
Median number of people living in the house (range)^a^	5 (2–15)	6 (2–19)	5 (2–19)***	7 (3–15)	5 (4–6)***
Median KMMS Part 1 score (IQR)	8 (4–12)	7 (3–15)	5 (2–8.5)***	15 (10–18)	8.5 (6–10)***

KMMS = Kimberley Mum’s Mood Scale. IQR = interquartile range.

* Significant at *P* = 0.009 compared to the group of antenatal participants.

** Significant at *P* = 0.02 compared to the group of participants who underwent the reference standard assessment.

*** Significant at *P* < 0.05 compared to the group clinically diagnosed with anxiety and/or depressive disorders.

^a^ Missing data: 3 antenatal and 3 postnatal; 2 with no anxiety/depression, 3 clinically diagnosed with anxiety/depression, and 1 missing the reference mental health assessment.

### Reliability and validity of KMMS in identifying women with anxiety or depressive disorders

Part 1 had good internal consistency reliability (Cronbach’s alpha = 0.89). For screening, the most clinically appropriate threshold based on ROC curves for Part 1 was greater than or equal to 9 and for overall KMMS risk was greater than or equal to moderate. Sensitivity, specificity, predictive values, area under the ROC curve and percentage correctly classified for these cut-points are listed in Table 2. Median Part 1 score for participants clinically diagnosed compared to those who were not was significantly higher (Table 1). Using these cut-points, for Part 1 and overall KMMS risk, the number of referrals for further review would have been 40 (41%) and 29 (30%) participants, respectively.

**Table 2:** Validity of KMMS to identify participants (n = 91) with a reference standard GP diagnosis of anxiety and/or depressive disorders.

	KMMS Part 1^a^	Risk based on KMMS Part 1 and 2 combined^b^
Sensitivity (95% CI)	87.0 (65.3–96.6)%	82.6 (60.5–94.3)%
Specificity (95% CI)	75.0 (62.8–84.3)%	86.8 (75.9–93.4)%
Positive predictive value	54.1%	67.9%
Negative predictive value	94.4%	93.7%
Area under the ROC curve (95% CI)	0.86 (0.77–0.94)	0.90 (0.83–0.97)
Correctly classified	78.0%	85.7%

KMMS = Kimberley Mum’s Mood Scale. GP = General Practitioner.

^a^ Diagnosis based on a blinded reference standard GP assessment using a KMMS Part 1 cut-point of 9.

^b^ Diagnosis based on a blinded reference standard GP assessment using an overall KMMS risk (Part 1 and 2 combined) cut-point of moderate.

Participants’ classification based on overall KMMS risk and clinical diagnosis is compared in Table 3. An overall KMMS risk of moderate or high detected everyone with clinically moderate or high severity anxiety and/or depression. Four participants were assessed as low KMMS risk but were diagnosed with low severity anxiety or depression by a GP within 24-hours of the KMMS administration. While these assessments appear discordant, the management plans developed with these participants using the KMMS were similar to those recommended by the GP. Another participant was referred to mental health services by the study personnel administering the KMMS and did not have the reference standard GP assessment.

**Table 3:** Cross tabulation of the classification of participants based on the overall KMMS risk and reference standard GP diagnosis.

Reference standard GP diagnosis	Risk based on KMMS Part 1 and Part 2				
	no	low	moderate	high	total
No anxiety or depressive disorders	**18**	**41**	9	0	68
Anxiety and/or depressive disorders					
Low severity	0	4	**4**	**1**	9
Moderate severity	0	0	**5**	**4**	9
High severity	0	0	**1**	**4**	5
Missing	1	4	0	1^a^	6
Total	19	49	19	10	97

KMMS = Kimberley Mum’s Mood Scale. GP = General Practitioner.

Bold typeface indicates concordant results based on a KMMS cut-point of moderate risk.

^a^ This participant was referred onto mental health services by the study personnel administering the KMMS as high risk of self-harm; the study GP was ill and unable to examine the participant.

### Acceptability of the KMMS

The feedback questionnaire was completed by 81 of 97 (84%) participants: median scores for Q1 (understood the questions), Q2 (felt comfortable) and Q5 (talking about childhood/home life) were 10, 10 and 8 (out of 10), respectively. There was very little negativity reported about the KMMS, and frequently women wanted other family members to take part (2–4 women per community/town). Of the 81 participants who completed the questionnaire 36 (44%) reported that the KMMS “was good”, “helpful”, and they “liked the questions”, as clear, “easy to answer” and to understand; 25 (31%) participants also said they liked “telling my story”.

“Able to talk about it because of the questions … Made it a little easier talking about my family life.”

(Participant 304)

“They explained everything to me clearly, so I could understand what they were asking me… I didn’t feel uncomfortable at all when they were asking me those questions.”

(Participant 508)

“Enjoyed talking about childhood … reminded me of a time I was really loved and felt safe–‘happy days’.”

(Participant 509)

“Being able to open up and talk about anything and feel safe … Being able to understand the questions was good.”

(Participant 711)

“Nothing was bad, the questions were asked in a good way…. Makes me realise, the past is in the past. But bringing up the past, thinking about the past, is a good thing. It is good to remember that I had a difficult time with my stepmother, but now she is like my mother she made a difference in my life.”

(Participant 802)

Participants reported feeling confident to tell clinicians if they felt that a question was too difficult and that they did not want to continue. Although 10 (12%) participants reported that during the discussion of difficult issues they were “not too happy” and felt uncomfortable, 3 (4%) said that while it was hard to talk about difficult aspects of their life it was “good to do”, and that it was important for health workers to understand their story. Only one (1%) participant reported that talking about their problems did not help.

I was hurt as a kid I don’t want to discuss this.

(Participant 902)

“Some of the questions were okay, some of the questions were not so good … It was alright to be able to talk aloud about. It made me feel uncomfortable but it was good to talk about it.”

(Participant 1037)

“Thinking about my childhood brought about anger but it is okay. Good for health workers to know about patient’s childhood to help [them] be in good health for baby; good physical and emotional health.”

(Participant 806)

“At the back of my head … just don’t like being asked questions because people can’t really do anything … Have told a lot of people my problems and it doesn’t help, just make you more miserable talking about it.”

*(Participant 509)*“

Even though I gave it [KMMS] half score it was okay. But sometimes it was hard because my big sisters grew us up.”

(Participant 1005)

Made me feel uncomfortable.

(Participant 1039)

Nine of 15 (60%) study personnel completed the questionnaire and eight (89%) reported that doing the KMMS was considerably or extremely useful and superior to the EPDS. They reported that Part 2 allowed them to ask questions that gave the woman an opportunity to express herself and resulted in a deeper understanding between them. They noticed that participants were highly resilient, and felt that the self-disclosure was empowering for the women and that they demonstrated a visible sense of improved wellbeing after their disclosure, with no expectation for clinicians to solve their problems.

“Well the KMMS is great compared to the EPDS. The EPDS creates distrust and questions before you get started and that confidence is really hard to get back.”

(Midwife and Child Health Nurse 2)

“The women understand more [using the KMMS] and that means something to them. The pictures make a really big difference, they make them identify with their own person within. The changing faces make them think about and help them say how they feel.”

(Midwife 9)

“I tried to use the EPDS but really just doesn’t work. The wording is complicated and I struggle with it. But the questions are great, that KMMS. Yeah I think it was really useful.”

(Midwife 4)

“It was really easy to use and the women really seemed to enjoy using it. The words were straightforward: and relevant to them. This was important because it means we have some credibility with the women. Not like the EPDS, I feel like it is a waste of my time, theirs too.”

(Midwife 3)

“I felt as though it seemed to make them feel strong. It gave them a chance to say how they’d overcome something, and I was able to compliment them on overcoming these problems. I could see they really enjoyed this, feeling like they are really strong women… The women got a lot out of it. They felt pleased they had told the story. I noticed that they were really beaming, it was amazing. They were beaming even more after talking to [the study GP]. She was fantastic.”

(Midwife 2)

“Many of the midwives and CHNs [child health nurses] didn’t know the participants. I didn’t, but the stories that were shared with me were amazing. I can’t believe the resilience of these women and what they’ve been through.”

(Midwife 4)

Study personnel also reported feeling distressed and powerless when hearing aspects of these stories. Some were troubled that they had not known about these experiences earlier, stating it would have altered their management plans. Extensive debriefing was required after participants divulged traumatic experiences. One study personnel felt that Part 2 was out of her scope of practice. Despite these challenges all but one said they fully understood the importance of the process, the more often they administered it the better they would get, and that with practice they would be confident and happy with routine use of the KMMS.

“It is hard to finish this and at the end of the day, it’s hard not to want to neck a bottle of wine to cope with [hearing their stories].”

(Midwife 4)

“Being pushed for time [in the routine hospital antenatal clinic] is an issue. I sometimes felt that I was passing her onto the GP and I was able to hurry her through.”

(Midwife 1)

“We really need to know who we can call to de-brief, have some ‘professional supervision’ so that we know that what we did was OK, how we might approach the next visit/follow-up; continued professional development, especially because there is frequently no one else. Also they have developed trust with us and we owe it to them to be the best that we can be. I am not talking about those who are mentally unwell, just those who are borderline and need someone safe to talk to.”

(*Midwife 4)*

“As a child health nurse I am not a counsellor so was a little bit worried to open a can of worms I could not put the lid back on.”

(Study personnel—anonymous online questionnaire)

“I needed to become familiar with the questions and follow through questioning—this would happen with more use of the tool.”

(Study personnel—anonymous online questionnaire)

The KMMS allowed generation of mutual respect, trust and understanding, with participants generally comfortable to say that they did not want to talk about it now.

“She [study personnel administering the tool] needs to know was right or wrong ways [to ask the question] so she can understand me more.”

(Participant 309)

“Being straight and honest. Being listened to and understanding one another.”

(Participant 703)

“They make you feel comfortable and it’s easy to give answers.”

(Participant 803)

“The prompters were good for difficult subjects that are too hard to broach. They were good because women chose to tell us as much or as little as they want to anyway.”

(Midwife and Child Health Nurse 2)

“I have much more respect for the women now, now that I know what they have put up with and that they are now proving that they are protective mothers and bringing up more children themselves and doing a good job.”

(Midwife 2)

“Because I know that I would be heard it was very good.”

(Participant 1034)

“You do not have to know the woman to have a trusting relationship.”

(Midwife 4)

Further quotes illustrating the acceptability of the KMMS are detailed in Figure E in S1 File.

## Discussion

Our study showed that the KMMS is an effective tool for identifying Kimberley Aboriginal perinatal women at risk of anxiety and depression. The KMMS appears to be much more readily accepted by clinicians than the EPDS and has potential to lead to more timely supportive interventions for Kimberley Aboriginal women such as the creation of a negotiated management plan. This extra support is likely to have significant benefits for patients [26, 27], their children and health care providers.

An overall KMMS risk of moderate or high detected everyone with clinically moderate or high severity anxiety or depression, suggesting that the KMMS is able to detect women who most urgently require immediate assistance. The sensitivity of the KMMS using a cut-point of moderate to detect anxiety and depressive disorders compares well with sensitivities reported in a systematic review for the commonly used EPDS cut-point of 9/10 for identifying minor and major postpartum depression diagnosed by a reference standard (83% v 59%-100%) [32]. The significantly higher median KMMS Part 1 scores of participants who were diagnosed with anxiety and/or depressive disorders compared to those who did not have anxiety or depression (15 v 5) is also consistent with research on the EPDS (18 v 4) [33].

Completing both parts of the KMMS is important. Part 1 scores should only be used to flag women at risk. Women did not always disclose important issues in Part 1, but these often came out during Part 2’s exploration of protective and risk factors, modifying the overall risk. It also forms the basis for a woman-centred and directed management plan. We recommend that women assessed as moderate or high risk are referred for mental health assessment by a GP or experienced mental health professional. Women assessed at high risk need an immediate increase in support/monitoring until appropriate referrals are activated.

Another important part of the KMMS is development of management plans for all women, even those at no or low risk. The management plans for the four participants who were assessed as low risk of anxiety and depression with the KMMS, but were subsequently diagnosed with low severity anxiety or depression, were similar to those recommended by the GP. Based on our recommendations these women would not be referred onto a GP for further assessment, however while the numbers are limited our study suggests that exploring protective and risk factors during Part 2’s mental health brief-intervention supported appropriate assistance for these women with low severity anxiety or depression.

We found the KMMS to be a culturally safe and non-judgemental tool. It was accepted by nearly all participants and, after being debriefed, by all but one of the study personnel. The guided questions created a safe space for participants to talk and an opportunity to decide if the clinician could cope with their reality and their world. Health care providers, who were generally not from the same cultural or socio-economic groups as the women being assessed, gained valuable insights into participants’ lived experiences and social, emotional and cultural well-being. Such understanding can increase engagement with a service and assist in early identification of mental health issues which is valuable in shaping supportive ongoing-care [34] to form the basis for a woman-centred and directed management plan. This process can also address issues such as capacity building of health services to ensure cultural competence, as well as highlighting the effect of social exclusion and institutionalised racism [35–38].

The strengths of our study include: blinded reference GP standard assessment (97% assessed within 24 hours of the KMMS); excellent participation (94%) and questionnaire response (84%) rates. Similar to the Kimberley Indigenous Cognitive Assessment [39], Part 1 is written in ‘Kimberley’ English, which can be readily translated for other Aboriginal and Torres Strait Islander populations [40].

The study was embedded into everyday remote health service practice which provided insights into how the KMMS can be implemented. Appropriate culturally safe training and support is crucial for successful routine use of KMMS. Kimberley health services have refined KMMS training and incorporated it into regional training programs [24]. In some situations an Aboriginal health worker with appropriate training could provide the KMMS assessment. However this is not always appropriate as they may be local to the community and related to the woman.

Another significant barrier to implementation of both parts of the KMMS is time constraints for health care providers and patients. We recommend that at least 30 minutes is allowed for administering the KMMS. This is in contrast to the process of administering the EPDS, which is often given to women as a tick box exercise, done alone whilst the health care worker is doing something else, such as weighing the baby. Patient discussion during the KMMS can bring up feelings that they may not have discussed previously. This can cause distress for both the patient and health care provider, which will then need to be managed and resolved by further referral for counselling.

The use of GPs to provide the reference standard assessment could be seen as a limitation of the study. However, study GPs were able to understand and respond within the local context, hence potentially more accurate than less locally experienced fly-in-fly-out mental health professionals, where suspicion, language, communication and cultural barriers may undermine diagnostic accuracy and participation rates [41, 42]. In terms of implementation, as 25% of participants were clinically diagnosed with anxiety and/or depressive disorders, it is neither practical nor desirable that such common problems are managed entirely by specialist services. GPs working for primary care services need to be engaged with these issues and comfortable with managing or sharing management in partnership with specialised mental health services.

The main limitations of our study are relatively small numbers, hence relatively wide 95% confidence intervals, and using a ‘convenience sample’. However, the sample size is consistent with similar validation studies including the initial Kimberley Indigenous Cognitive Assessment instrument [39] study. Study personnel wanted to make sure women they thought were at risk were offered a chance to take part, and thus our sample may not be representative of the Kimberley Aboriginal perinatal population. A small number of participants also did not receive the reference standard GP assessment. Despite these limitations, as KMMS’ diagnostic accuracy and acceptability is better than current practice Kimberley regional guidelines now recommend using the KMMS as an alternative to the EPDS during the perinatal period for Aboriginal women. It is important that the KMMS is re-evaluated in a larger Kimberley population during real world implementation, and to test for applicability in other remote regions to inform recommendations for wider use. To do this we will use a similar process to that used with the Kimberley Indigenous Cognitive Assessment instrument, which was developed and initially validated in the Kimberley with 70 Aboriginal participants [39], then re-evaluated in the Kimberley with a larger group (363 Aboriginal participants), and adapted for use and validated in other Australian locations [40, 43, 44].

## Conclusion

There are linguistic, cultural and practical reasons why a different approach to screening remote Aboriginal populations is required. Our study shows that adopting the KMMS in the Kimberley, and with suitable modifications elsewhere in remote Australia may improve perinatal mental health screening and support timely and relevant intervention. A similar approach could also be used in other countries where there are significant cultural differences between health care providers and patients.

## Supporting Information

S1 File Kimberley Mum’s Mood Scale (KMMS) (**Fig A**). Validating the Kimberley Mum’s Mood Scale (KMMS) GP Data Collection Form (**Fig B**) Validating the Kimberley Mum’s Mood Scale (KMMS) Participant Feedback Form (**Fig C**). KMMS Study Personnel Online Questionnaire (**Fig D**). Further quotes illustrating the acceptability of the KMMS (**Fig E**).
(ZIP)
