# Supplementary material for: Validity and Acceptability of Kimberley Mum’s Mood Scale to Screen for Perinatal Anxiety and Depression in Remote Aboriginal Health Care Settings
Source: PLoS One. 2017 Jan 30;12(1):e0168969. doi: 10.1371/journal.pone.0168969 (PMC5279756; doi:10.1371/journal.pone.0168969)
Supplement: S1 File — Kimberley Mum’s Mood Scale (KMMS) (Fig A). Validating the Kimberley Mum’s Mood Scale (KMMS) GP Data Collection Form (Fig B) Validating the Kimberley Mum’s Mood Scale (KMMS) Participant Feedback Form (Fig C). KMMS Study Personnel Online Questionnaire (Fig D). Further quotes illustrating the acceptability of the KMMS (Fig E). (ZIP) [file pone.0168969.s001.zip › S1 Appendix Fig E - quotes R1.docx]

## S1 Appendix Fig E. Further quotes illustrating the acceptability of the KMMS

### Good to talk about it

Good to have someone to talk to and listen to me. *(Participant 305)*

Found the questions easy to answer. *(Participant 306)*

All the questions was very good. I like it. *(Participant 312)*

It was very good because I like telling my life story. *(Participant 410)*

Pictures were alright. *(Participant 510)*

I felt a bit shy… but it was good to answer those questions. *(Participant 702)*

Being able to speak about the problem I have with someone. *(Participant 709)*

It was good to talk about it, it was good to get it off my chest. *(Participant 801)*

Found it easy talking about my childhood. *(Participant 803)*

Grew up in good home and safe environment. If I didn’t grow up in a good home I may not have felt so comfortable. *(Participant 901)*

Very helpful and it was all put in a way that I could understand. *(Participant 1001)*

Made sense and was helpful. *(Participant 1032)*

### The KMMS is great compared to the EPDS

The questions were easy. I really think the women enjoy using it. They seemed to like pictures and the faces. Faces meant something to them, you know it’s helped them translate how they feel also. *(Midwife 4)*

They can see the pictures and they seem to really like that. It’s quicker to use than the Edinburgh depression scale because it requires no explanation or re-defining, it makes sense to them. *(Midwife and Child Health Nurse 2)*

The format [of KMMS] is really good, the way you roll out an assessment with Part 1, it starts with the pictures and the simple wording; the women really lighten up and enjoyed doing this first page. And the wording is put in a more positive way, not negative statements. *(Midwife 1)*

It was so good to have something so concrete to explore with women the things we know are distressing for them. They choose the priority to discuss and disclose with us. The EPDS is just a tick box, and this is limiting and workers don’t really talk to the participant. With the EPDS if they are in the scope of normal, then as staff we don’t expand or explore, whereas the focus is not on the number so much and you don’t have that ‘escape clause’, you have to do Part 2, so you need to explore these issues. The KMMS allows much less room for ‘escape’ that is a good thing. But the midwife still needs to have a trusting attitude. *(Midwife 1)*

I can’t use the EPDS according to protocol. I have to use it and adapted it, which is supposed to, I guess change its validity. It is not meant to be administered that way. And the truth is, I often don’t use it because it’s just not appropriate a lot of the time. But Part 1 of the KMMS is so much easier … The Part 1 is really good that didn’t take as long as I thought it would, certainly not as long as the Edinburgh Postnatal Depression Scale. *(Midwife 3)*

### Women opened up much more than they had before

Women really seemed to love ‘telling their story’. Telling the story is critically different to ‘gathering a history’ even though they amounted to the same thing in the end … I wish we had more access to the really young women [<18 year olds], I really think that being able to tell their story in safety would have a real impact on them at the beginning of their role as a new mum especially in this context. *(Midwife 1)*

[The women] seem to like talking about their confidence, you know, what had gone wrong [for them] and how they overcame it. I felt as though it seemed to make them feel stronger. It gave them a chance to say how they had overcome something, and I was able to compliment them on overcoming these problems. I could see that they really enjoyed this feeling, like they are really strong women. *(Midwife and Child Health Nurse 2)*

I picked up that these women have the most amazing amount of resilience and they have no expectation that you will ‘do’ anything more than just hold the safe place for her. (*Midwife 5)*

The resilience in these women when they have been through so much, for me just hearing about how much, and I wanted to cry when they told me things. And then just hearing it made me feel shocked to the core with some women, and I just think ‘how strong are you to be getting this every day’, and I’m just hearing your story and I’m broken up about it. *(Midwife 4)*

### Troubled by the stories they heard

I felt like it was too deep, too involved and oh my God! I haven’t had enough training in all these areas and am not a social worker or a psychologist. *(Midwife 2)*

As this was new to me at first it felt awkward until you developed your own style and phrasing for the questions. *(Study personnel – anonymous online questionnaire)*

Just more training for us before we do it please. *(Study personnel – anonymous online questionnaire)*

### Generation of mutual respect, trust and understanding

Questions made me think and thinking was good for me because you understand me better now. *(Participant 1007)*

The nurses made me feel really comfortable to talk in front of her. *(Participant 1002)*

The pictures make a really big difference because they just look at the pictures and know how they feel. They need to trust the person that they can sit down with and do that document with. *(Child Health Nurse 1)*
