# Supplementary figures and images for: Validity and Acceptability of Kimberley Mum’s Mood Scale to Screen for Perinatal Anxiety and Depression in Remote Aboriginal Health Care Settings
Source: PLoS One. 2017 Jan 30;12(1):e0168969. doi: 10.1371/journal.pone.0168969 (PMC5279756; doi:10.1371/journal.pone.0168969)

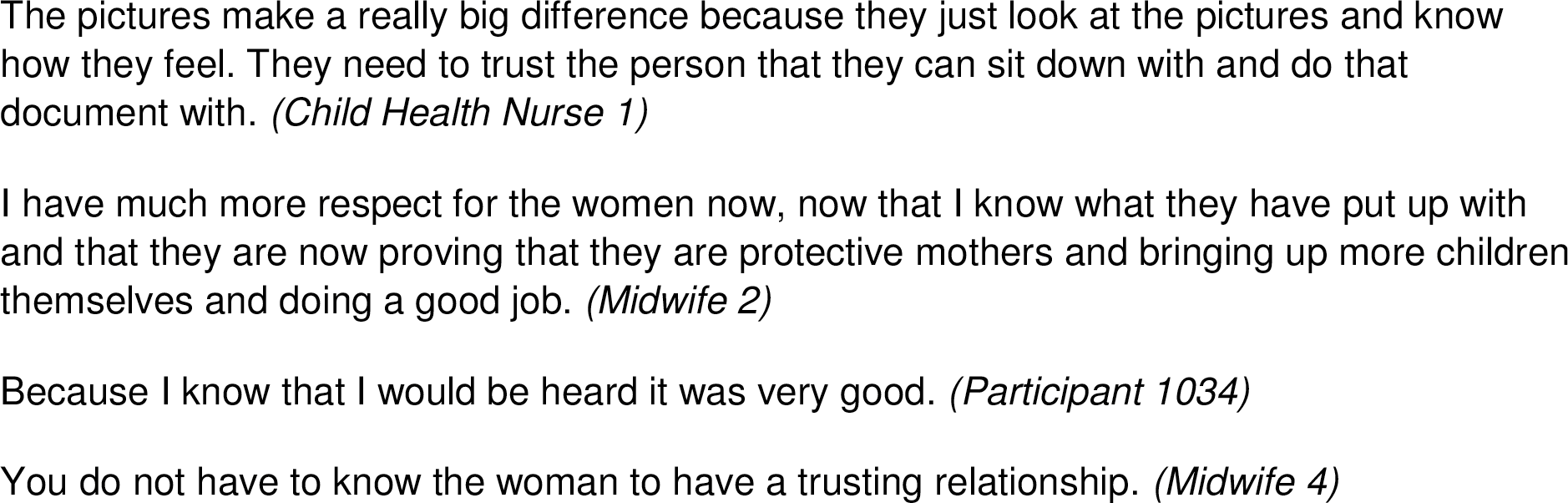

Supplement: S1 File — Kimberley Mum’s Mood Scale (KMMS) (Fig A). Validating the Kimberley Mum’s Mood Scale (KMMS) GP Data Collection Form (Fig B) Validating the Kimberley Mum’s Mood Scale (KMMS) Participant Feedback Form (Fig C). KMMS Study Personnel Online Questionnaire (Fig D). Further quotes illustrating the acceptability of the KMMS (Fig E). (ZIP) [file pone.0168969.s001.zip › S1 Appendix Fig E - 5.tif]

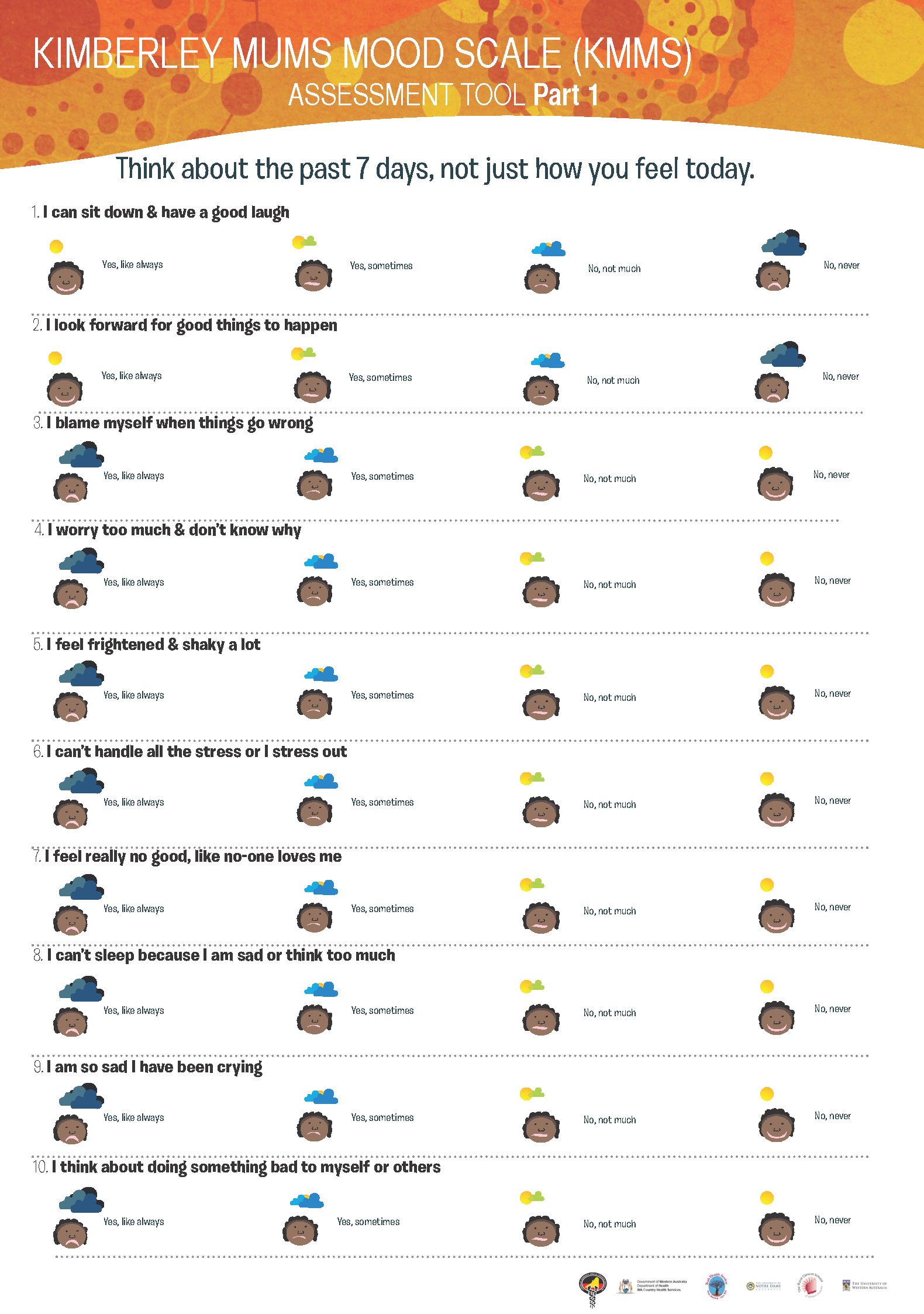

Supplement: S1 File — Kimberley Mum’s Mood Scale (KMMS) (Fig A). Validating the Kimberley Mum’s Mood Scale (KMMS) GP Data Collection Form (Fig B) Validating the Kimberley Mum’s Mood Scale (KMMS) Participant Feedback Form (Fig C). KMMS Study Personnel Online Questionnaire (Fig D). Further quotes illustrating the acceptability of the KMMS (Fig E). (ZIP) [file pone.0168969.s001.zip › S1 Appendix Fig A - 1.tif]

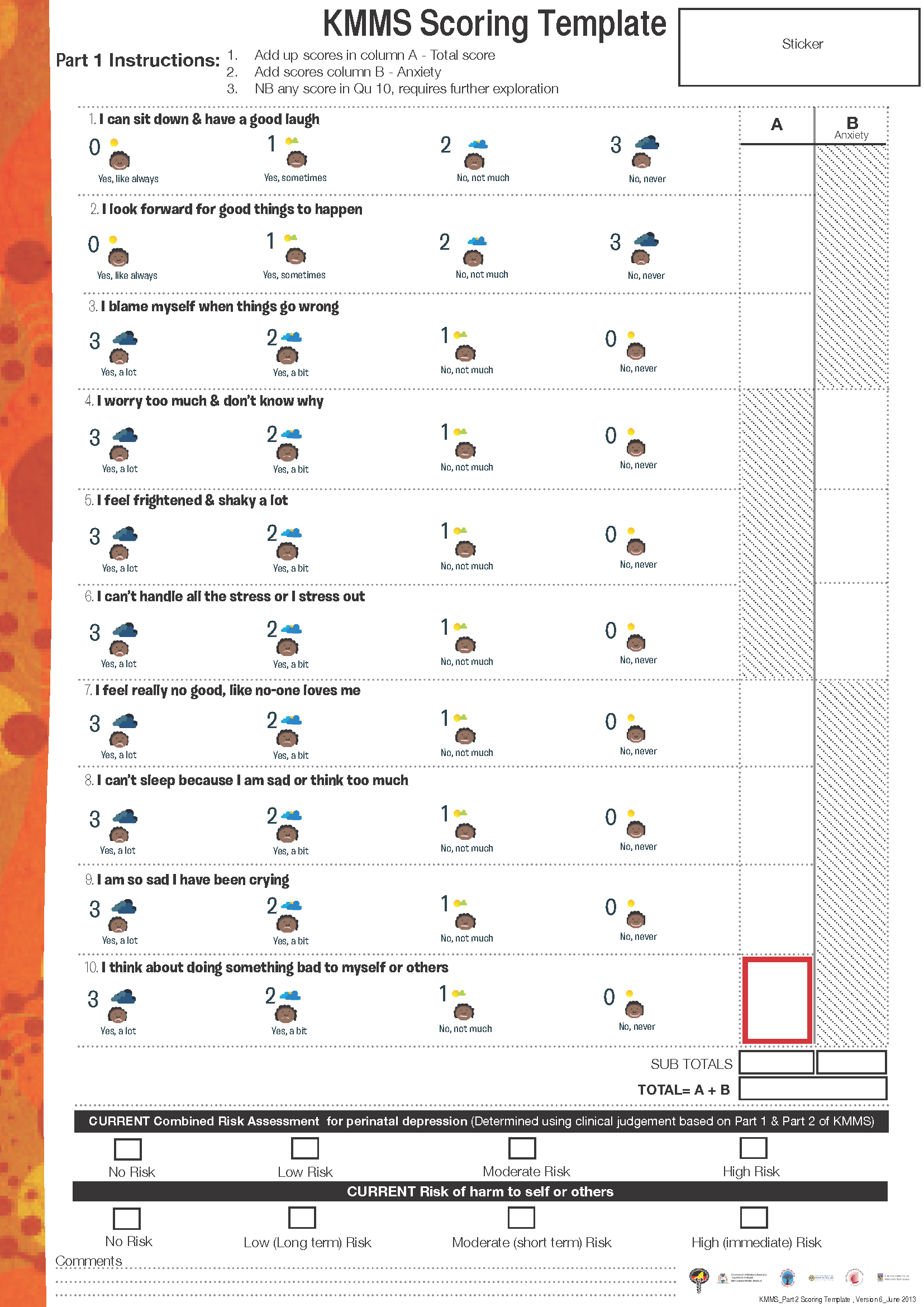

Supplement: S1 File — Kimberley Mum’s Mood Scale (KMMS) (Fig A). Validating the Kimberley Mum’s Mood Scale (KMMS) GP Data Collection Form (Fig B) Validating the Kimberley Mum’s Mood Scale (KMMS) Participant Feedback Form (Fig C). KMMS Study Personnel Online Questionnaire (Fig D). Further quotes illustrating the acceptability of the KMMS (Fig E). (ZIP) [file pone.0168969.s001.zip › S1 Appendix Fig A - 2.tif]

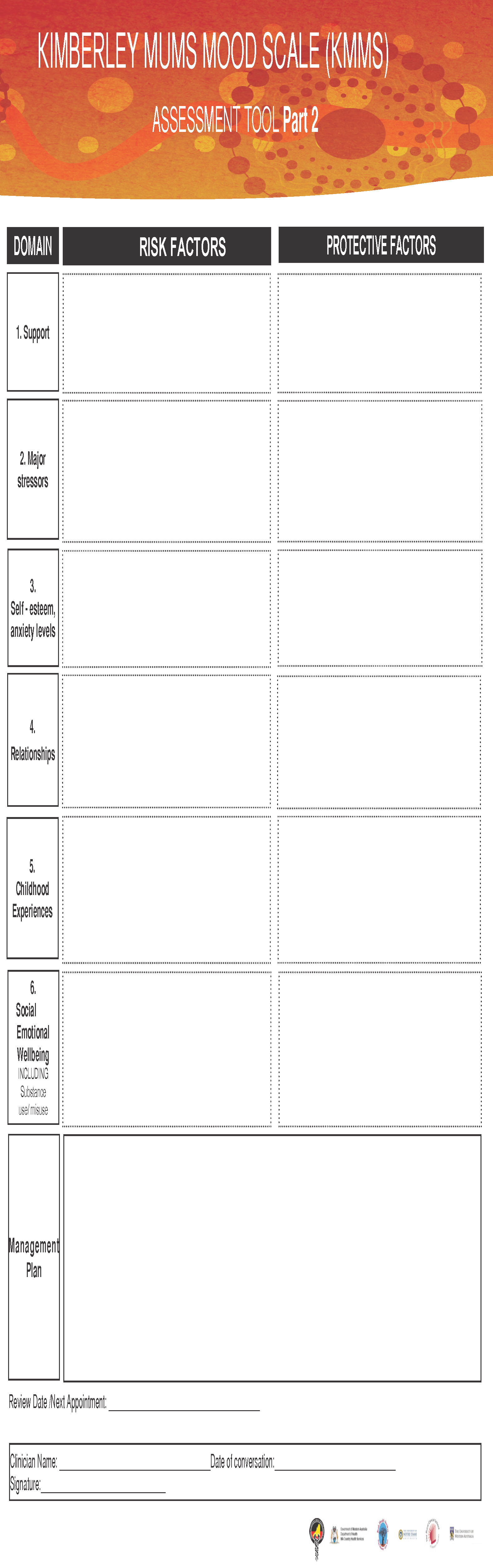

Supplement: S1 File — Kimberley Mum’s Mood Scale (KMMS) (Fig A). Validating the Kimberley Mum’s Mood Scale (KMMS) GP Data Collection Form (Fig B) Validating the Kimberley Mum’s Mood Scale (KMMS) Participant Feedback Form (Fig C). KMMS Study Personnel Online Questionnaire (Fig D). Further quotes illustrating the acceptability of the KMMS (Fig E). (ZIP) [file pone.0168969.s001.zip › S1 Appendix Fig A - 3.tif]

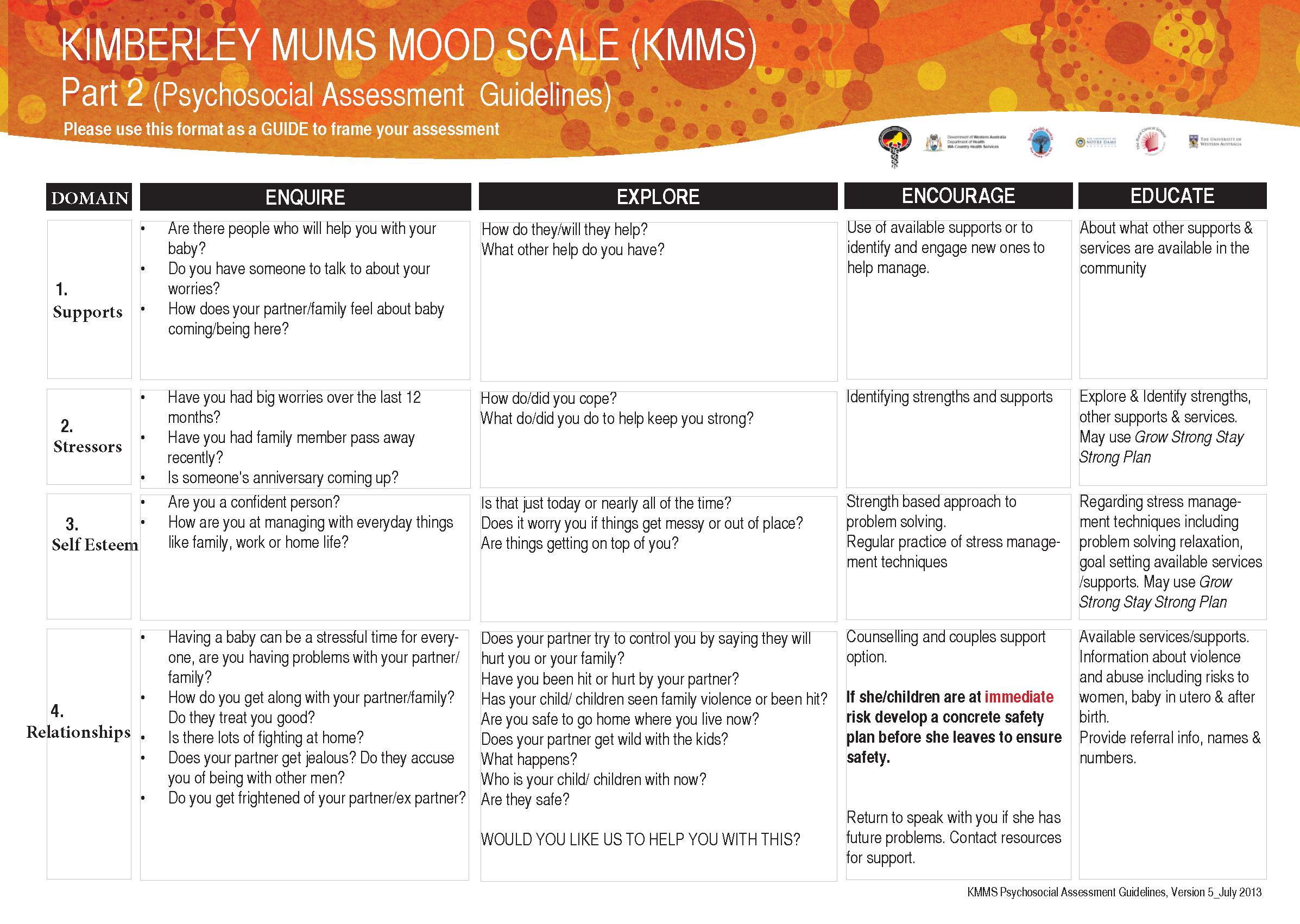

Supplement: S1 File — Kimberley Mum’s Mood Scale (KMMS) (Fig A). Validating the Kimberley Mum’s Mood Scale (KMMS) GP Data Collection Form (Fig B) Validating the Kimberley Mum’s Mood Scale (KMMS) Participant Feedback Form (Fig C). KMMS Study Personnel Online Questionnaire (Fig D). Further quotes illustrating the acceptability of the KMMS (Fig E). (ZIP) [file pone.0168969.s001.zip › S1 Appendix Fig A - 4.tif]

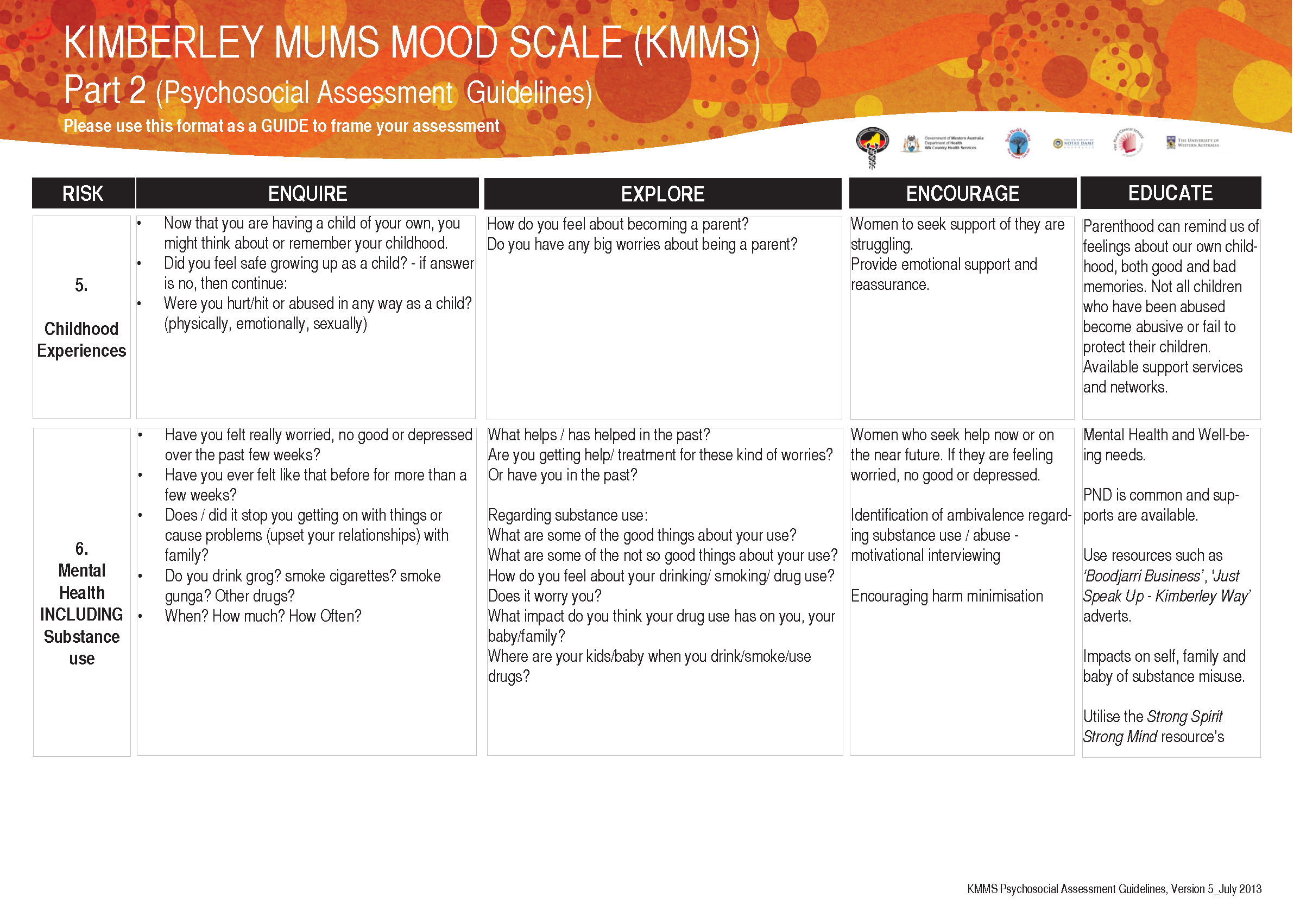

Supplement: S1 File — Kimberley Mum’s Mood Scale (KMMS) (Fig A). Validating the Kimberley Mum’s Mood Scale (KMMS) GP Data Collection Form (Fig B) Validating the Kimberley Mum’s Mood Scale (KMMS) Participant Feedback Form (Fig C). KMMS Study Personnel Online Questionnaire (Fig D). Further quotes illustrating the acceptability of the KMMS (Fig E). (ZIP) [file pone.0168969.s001.zip › S1 Appendix Fig A - 5.tif]

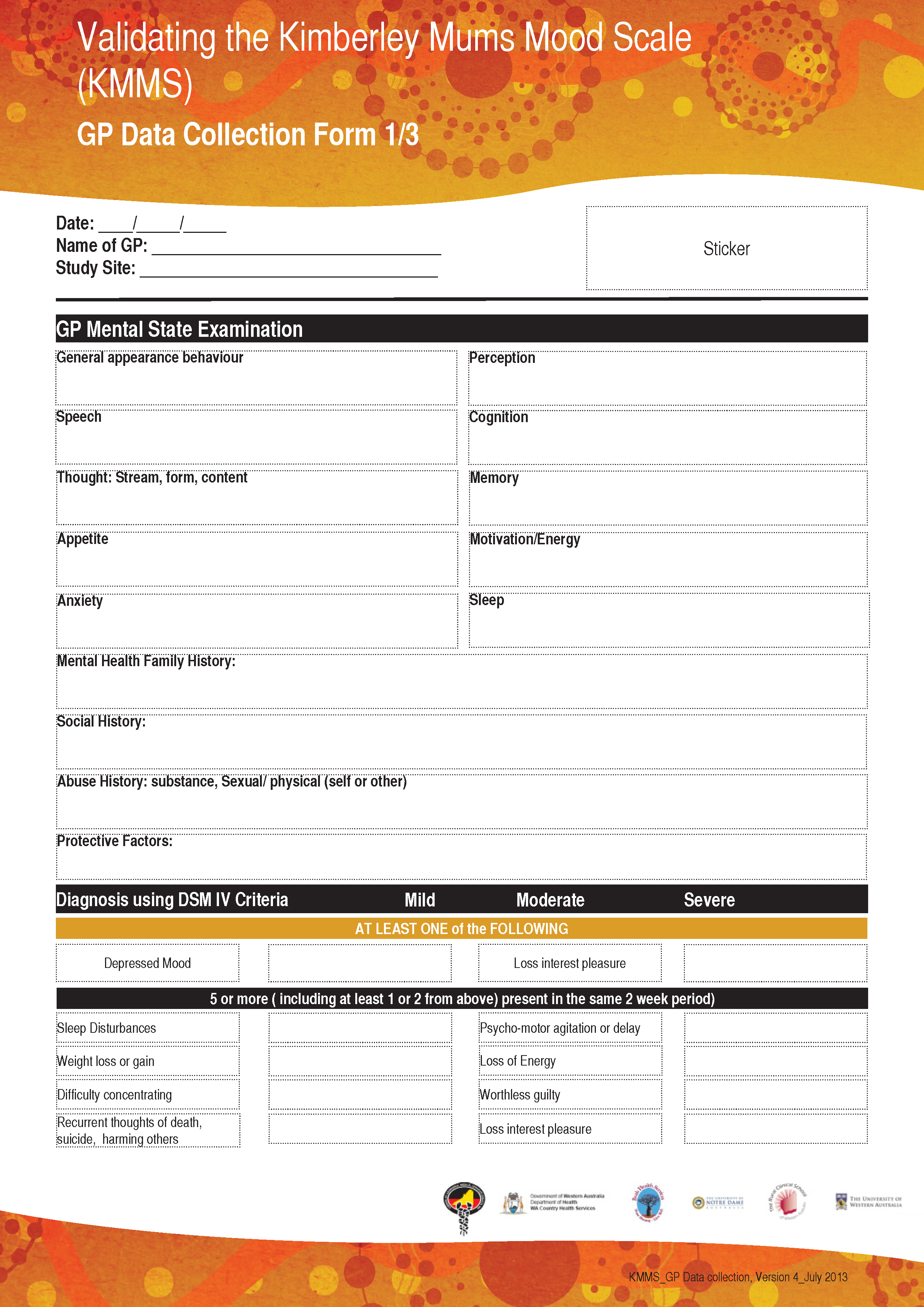

Supplement: S1 File — Kimberley Mum’s Mood Scale (KMMS) (Fig A). Validating the Kimberley Mum’s Mood Scale (KMMS) GP Data Collection Form (Fig B) Validating the Kimberley Mum’s Mood Scale (KMMS) Participant Feedback Form (Fig C). KMMS Study Personnel Online Questionnaire (Fig D). Further quotes illustrating the acceptability of the KMMS (Fig E). (ZIP) [file pone.0168969.s001.zip › S1 Appendix Fig B - 1.tif]

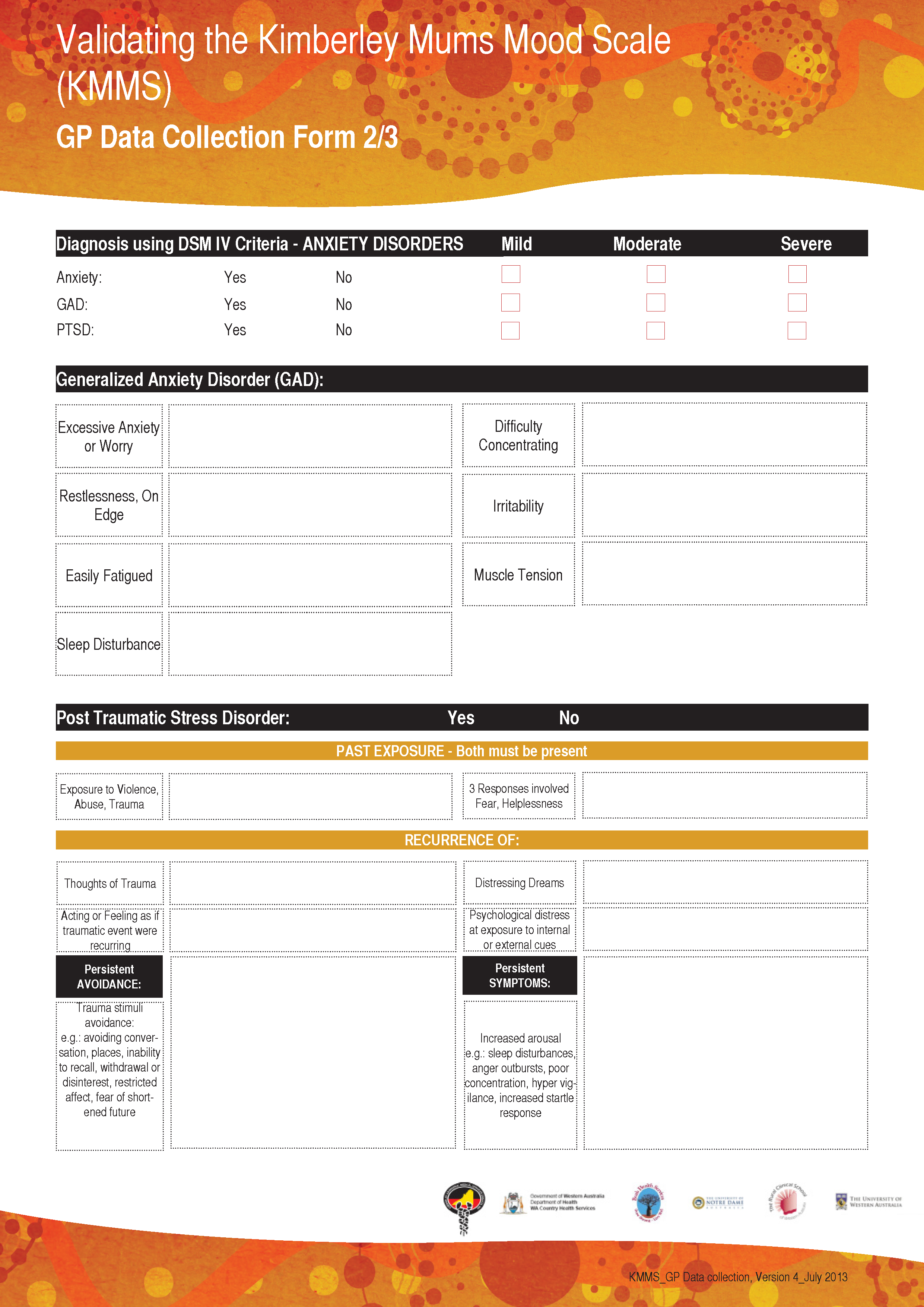

Supplement: S1 File — Kimberley Mum’s Mood Scale (KMMS) (Fig A). Validating the Kimberley Mum’s Mood Scale (KMMS) GP Data Collection Form (Fig B) Validating the Kimberley Mum’s Mood Scale (KMMS) Participant Feedback Form (Fig C). KMMS Study Personnel Online Questionnaire (Fig D). Further quotes illustrating the acceptability of the KMMS (Fig E). (ZIP) [file pone.0168969.s001.zip › S1 Appendix Fig B - 2.tif]

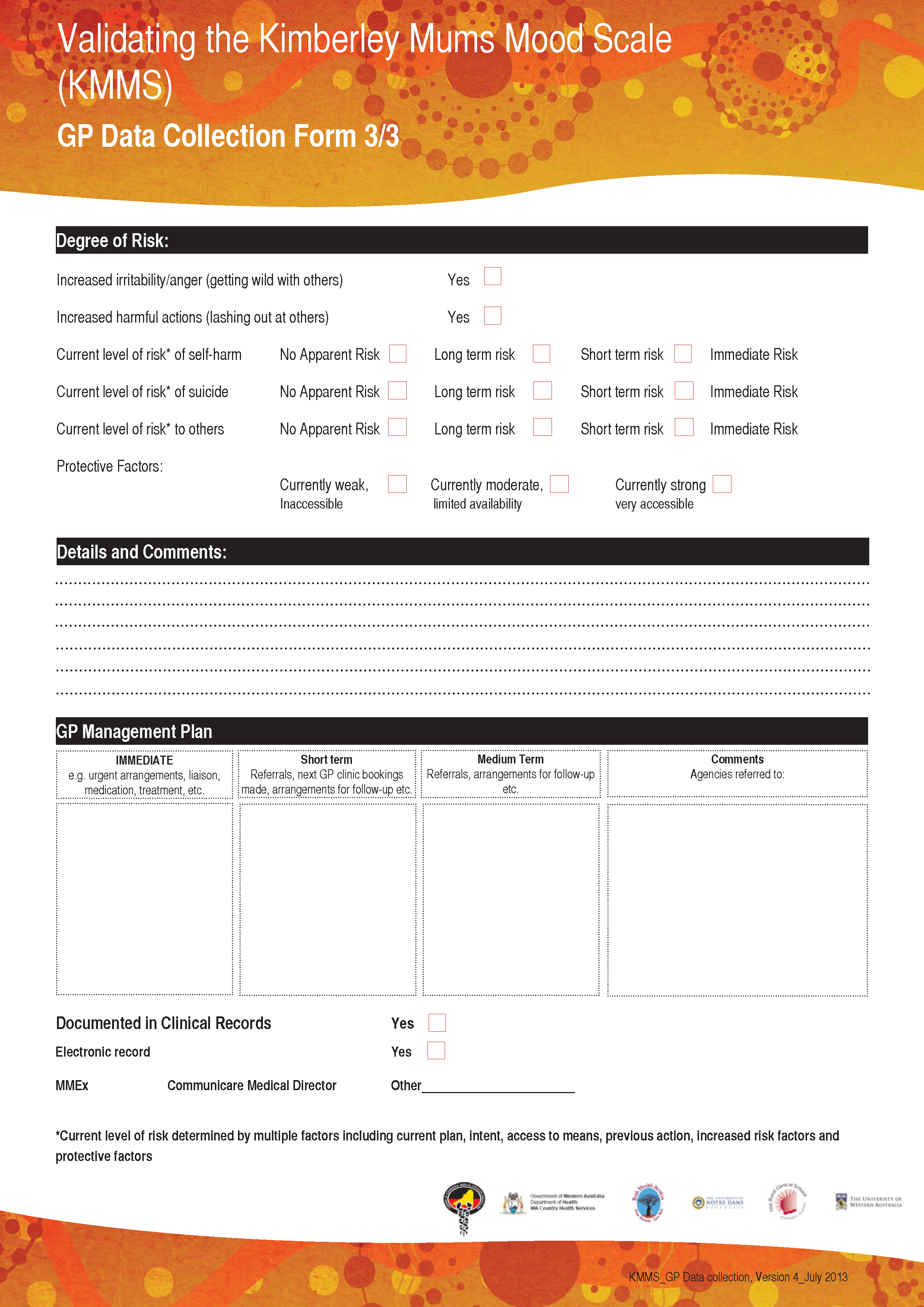

Supplement: S1 File — Kimberley Mum’s Mood Scale (KMMS) (Fig A). Validating the Kimberley Mum’s Mood Scale (KMMS) GP Data Collection Form (Fig B) Validating the Kimberley Mum’s Mood Scale (KMMS) Participant Feedback Form (Fig C). KMMS Study Personnel Online Questionnaire (Fig D). Further quotes illustrating the acceptability of the KMMS (Fig E). (ZIP) [file pone.0168969.s001.zip › S1 Appendix Fig B - 3.tif]

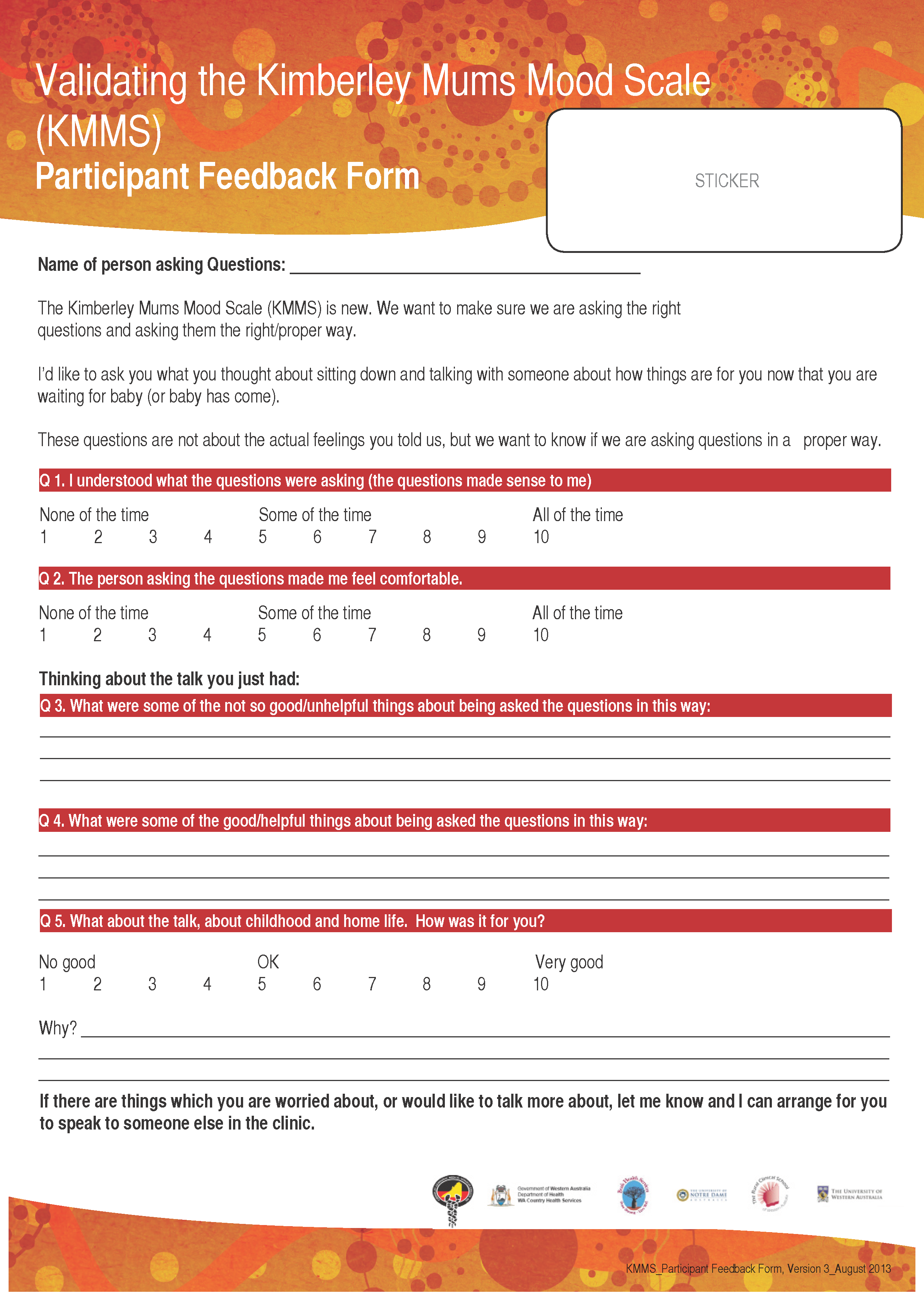

Supplement: S1 File — Kimberley Mum’s Mood Scale (KMMS) (Fig A). Validating the Kimberley Mum’s Mood Scale (KMMS) GP Data Collection Form (Fig B) Validating the Kimberley Mum’s Mood Scale (KMMS) Participant Feedback Form (Fig C). KMMS Study Personnel Online Questionnaire (Fig D). Further quotes illustrating the acceptability of the KMMS (Fig E). (ZIP) [file pone.0168969.s001.zip › S1 Appendix Fig C.tif]

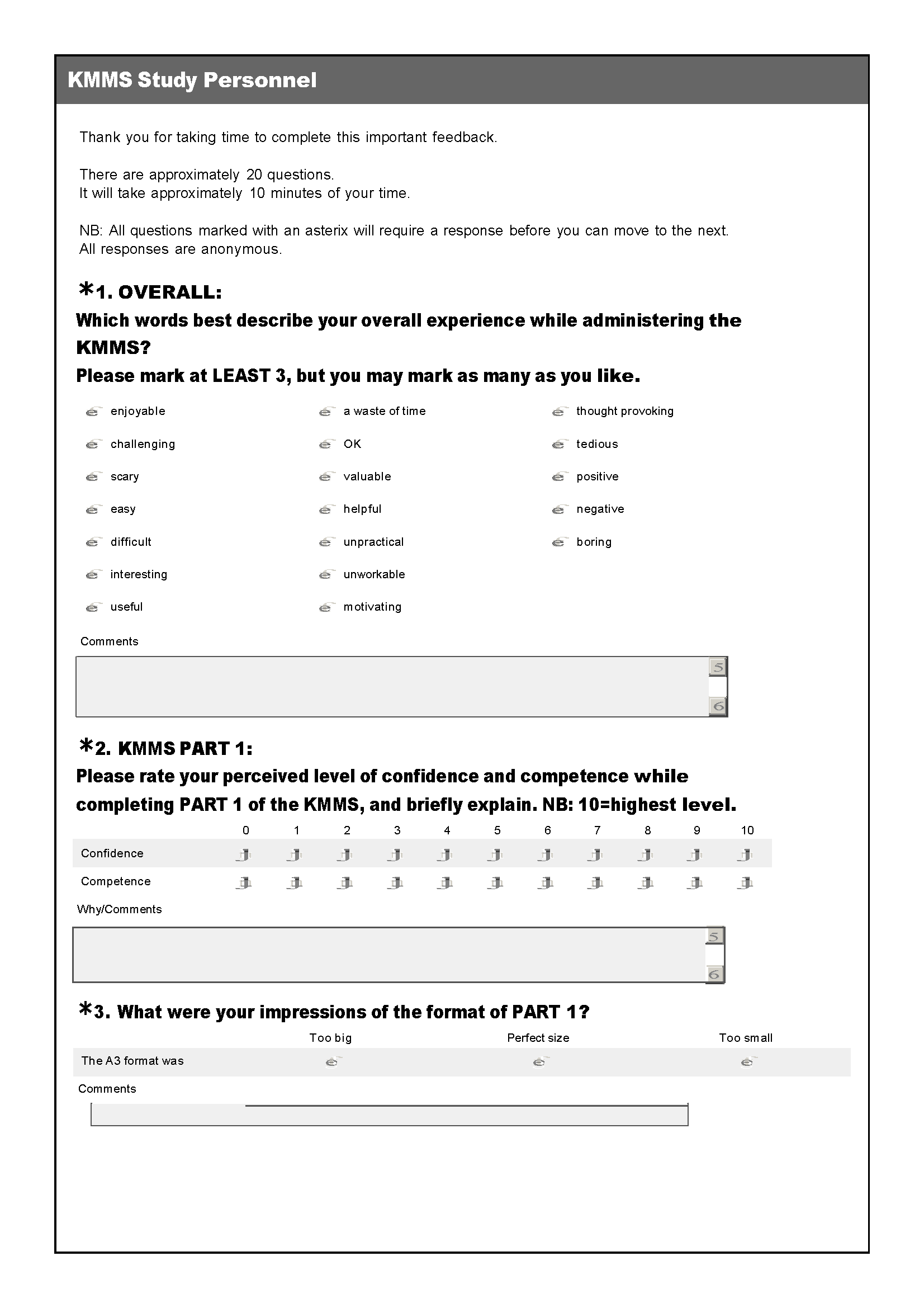

Supplement: S1 File — Kimberley Mum’s Mood Scale (KMMS) (Fig A). Validating the Kimberley Mum’s Mood Scale (KMMS) GP Data Collection Form (Fig B) Validating the Kimberley Mum’s Mood Scale (KMMS) Participant Feedback Form (Fig C). KMMS Study Personnel Online Questionnaire (Fig D). Further quotes illustrating the acceptability of the KMMS (Fig E). (ZIP) [file pone.0168969.s001.zip › S1 Appendix Fig D - 1.tif]

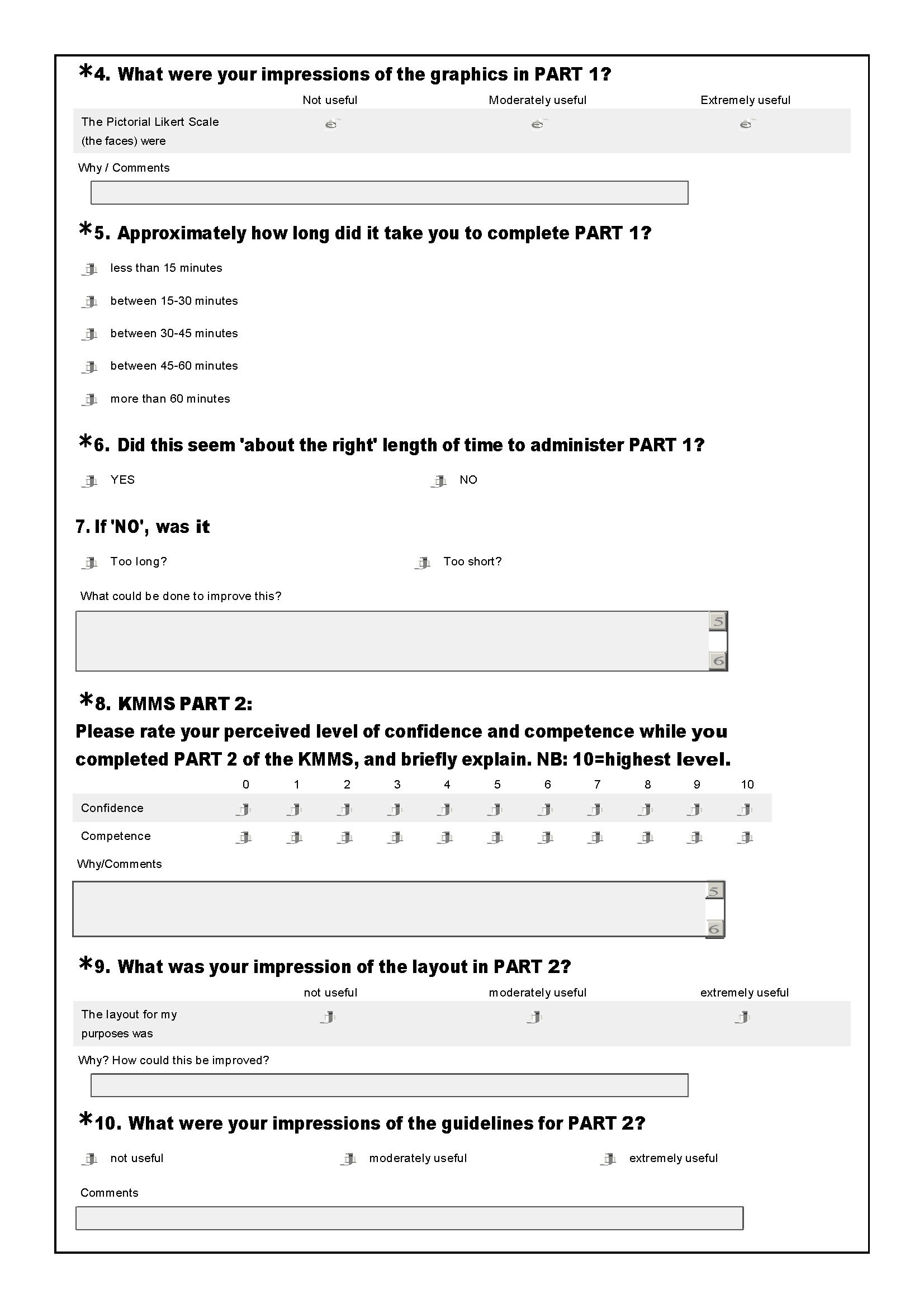

Supplement: S1 File — Kimberley Mum’s Mood Scale (KMMS) (Fig A). Validating the Kimberley Mum’s Mood Scale (KMMS) GP Data Collection Form (Fig B) Validating the Kimberley Mum’s Mood Scale (KMMS) Participant Feedback Form (Fig C). KMMS Study Personnel Online Questionnaire (Fig D). Further quotes illustrating the acceptability of the KMMS (Fig E). (ZIP) [file pone.0168969.s001.zip › S1 Appendix Fig D - 2.tif]

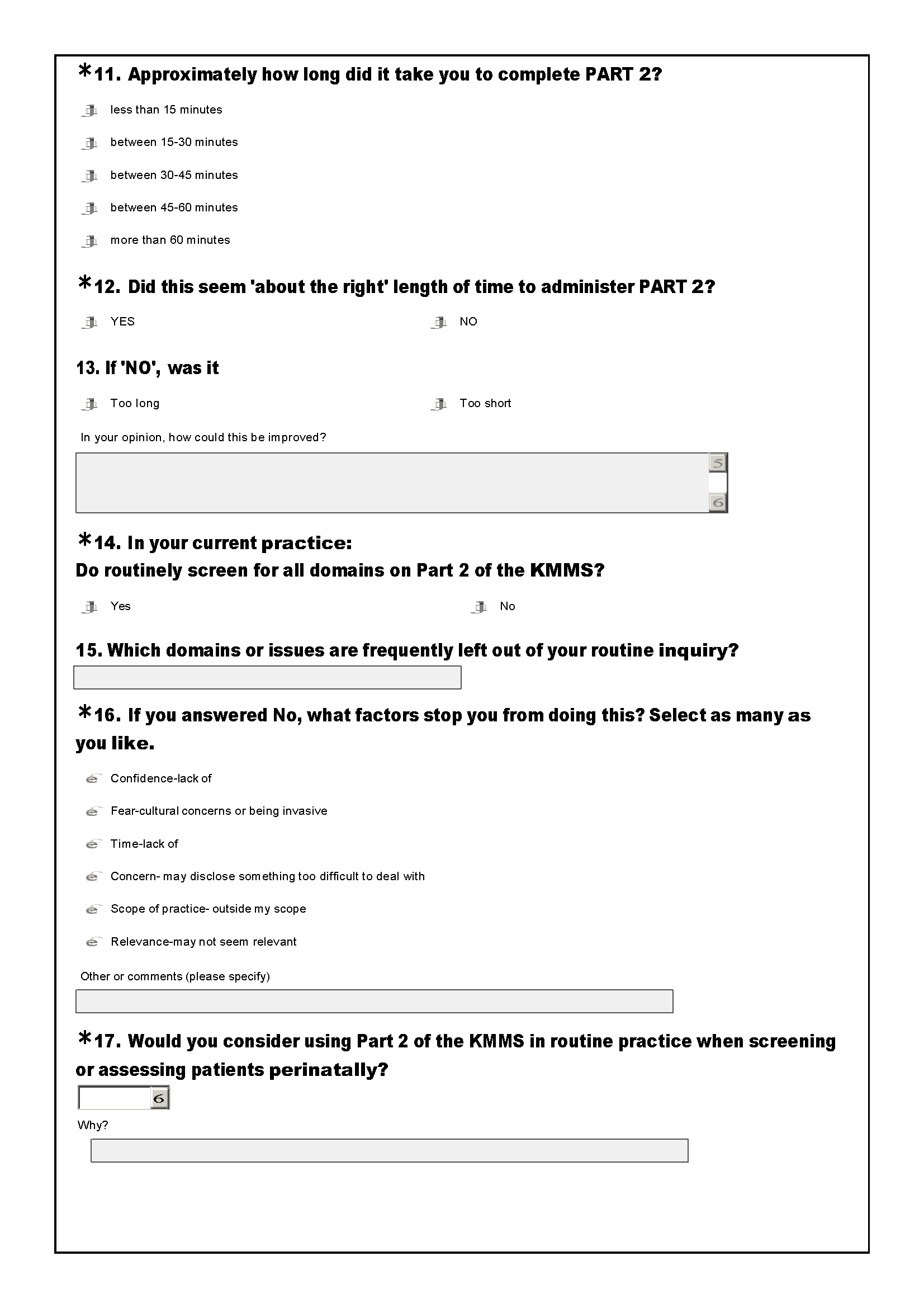

Supplement: S1 File — Kimberley Mum’s Mood Scale (KMMS) (Fig A). Validating the Kimberley Mum’s Mood Scale (KMMS) GP Data Collection Form (Fig B) Validating the Kimberley Mum’s Mood Scale (KMMS) Participant Feedback Form (Fig C). KMMS Study Personnel Online Questionnaire (Fig D). Further quotes illustrating the acceptability of the KMMS (Fig E). (ZIP) [file pone.0168969.s001.zip › S1 Appendix Fig D - 3.tif]

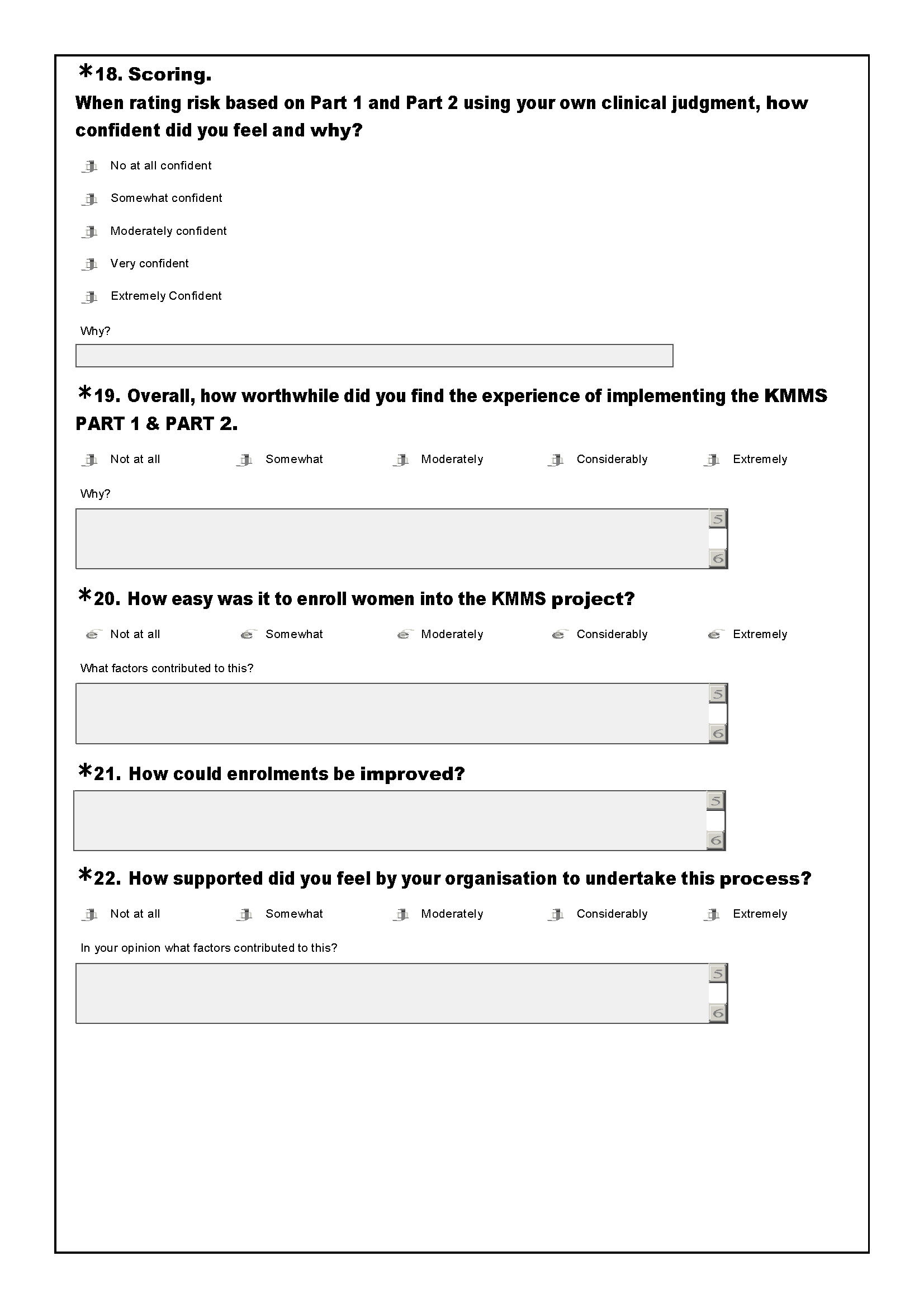

Supplement: S1 File — Kimberley Mum’s Mood Scale (KMMS) (Fig A). Validating the Kimberley Mum’s Mood Scale (KMMS) GP Data Collection Form (Fig B) Validating the Kimberley Mum’s Mood Scale (KMMS) Participant Feedback Form (Fig C). KMMS Study Personnel Online Questionnaire (Fig D). Further quotes illustrating the acceptability of the KMMS (Fig E). (ZIP) [file pone.0168969.s001.zip › S1 Appendix Fig D - 4.tif]

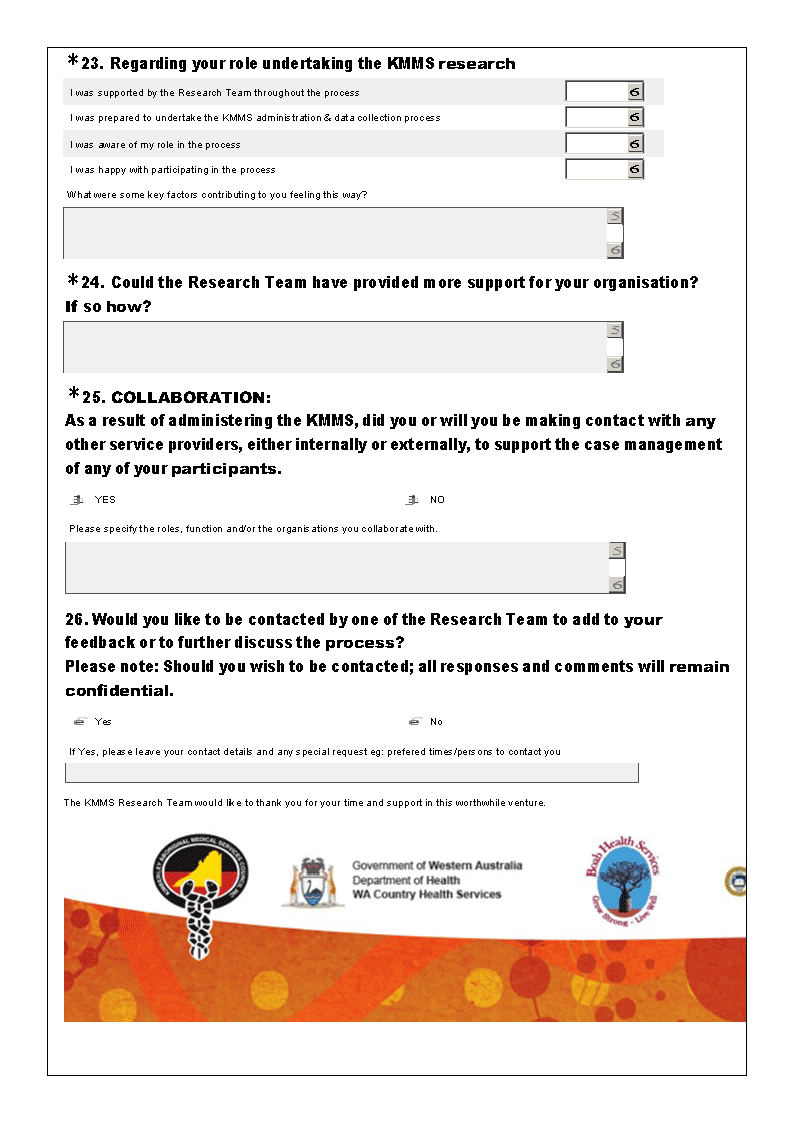

Supplement: S1 File — Kimberley Mum’s Mood Scale (KMMS) (Fig A). Validating the Kimberley Mum’s Mood Scale (KMMS) GP Data Collection Form (Fig B) Validating the Kimberley Mum’s Mood Scale (KMMS) Participant Feedback Form (Fig C). KMMS Study Personnel Online Questionnaire (Fig D). Further quotes illustrating the acceptability of the KMMS (Fig E). (ZIP) [file pone.0168969.s001.zip › S1 Appendix Fig D - 5.tif]

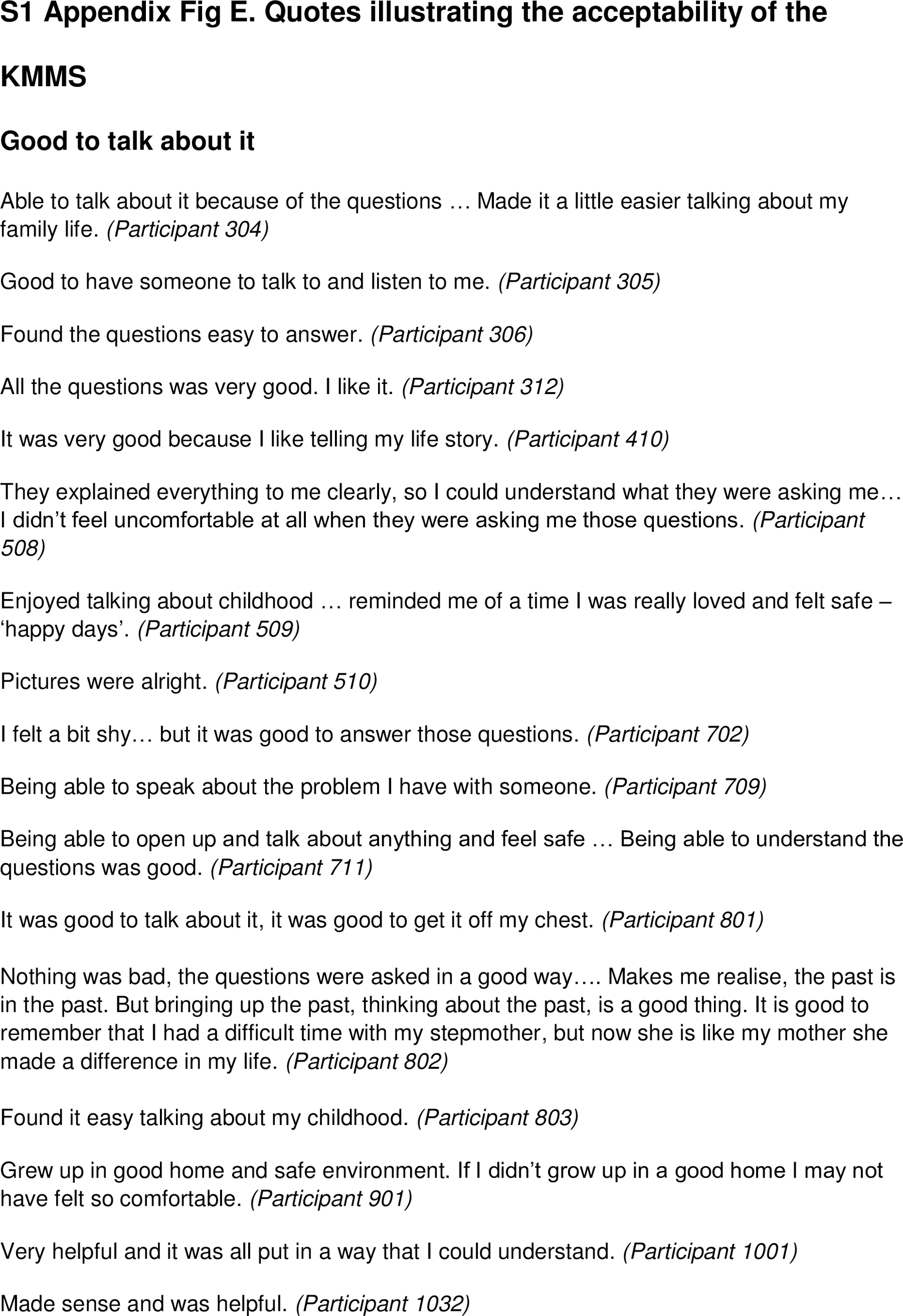

Supplement: S1 File — Kimberley Mum’s Mood Scale (KMMS) (Fig A). Validating the Kimberley Mum’s Mood Scale (KMMS) GP Data Collection Form (Fig B) Validating the Kimberley Mum’s Mood Scale (KMMS) Participant Feedback Form (Fig C). KMMS Study Personnel Online Questionnaire (Fig D). Further quotes illustrating the acceptability of the KMMS (Fig E). (ZIP) [file pone.0168969.s001.zip › S1 Appendix Fig E - 1.tif]

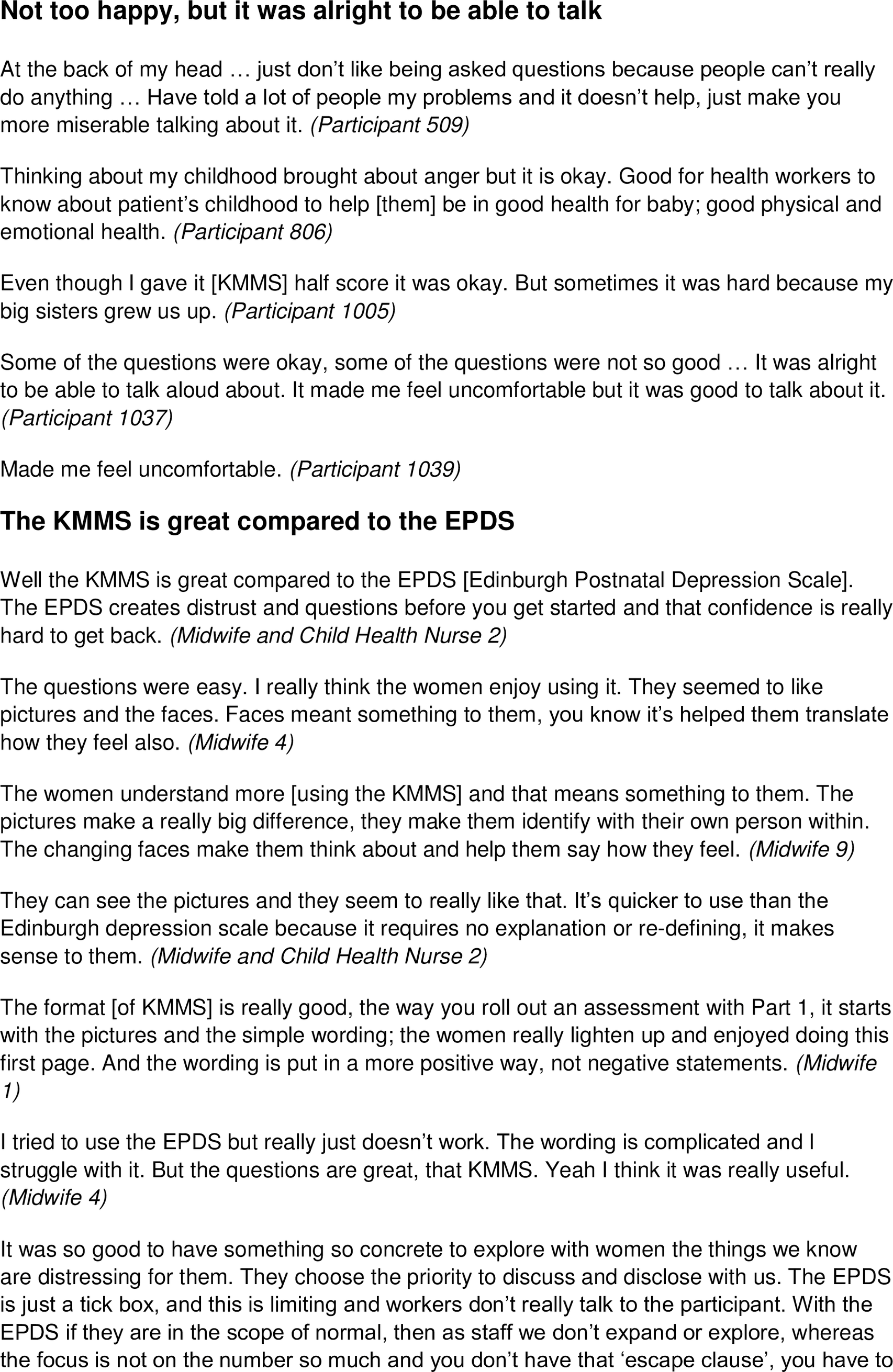

Supplement: S1 File — Kimberley Mum’s Mood Scale (KMMS) (Fig A). Validating the Kimberley Mum’s Mood Scale (KMMS) GP Data Collection Form (Fig B) Validating the Kimberley Mum’s Mood Scale (KMMS) Participant Feedback Form (Fig C). KMMS Study Personnel Online Questionnaire (Fig D). Further quotes illustrating the acceptability of the KMMS (Fig E). (ZIP) [file pone.0168969.s001.zip › S1 Appendix Fig E - 2.tif]

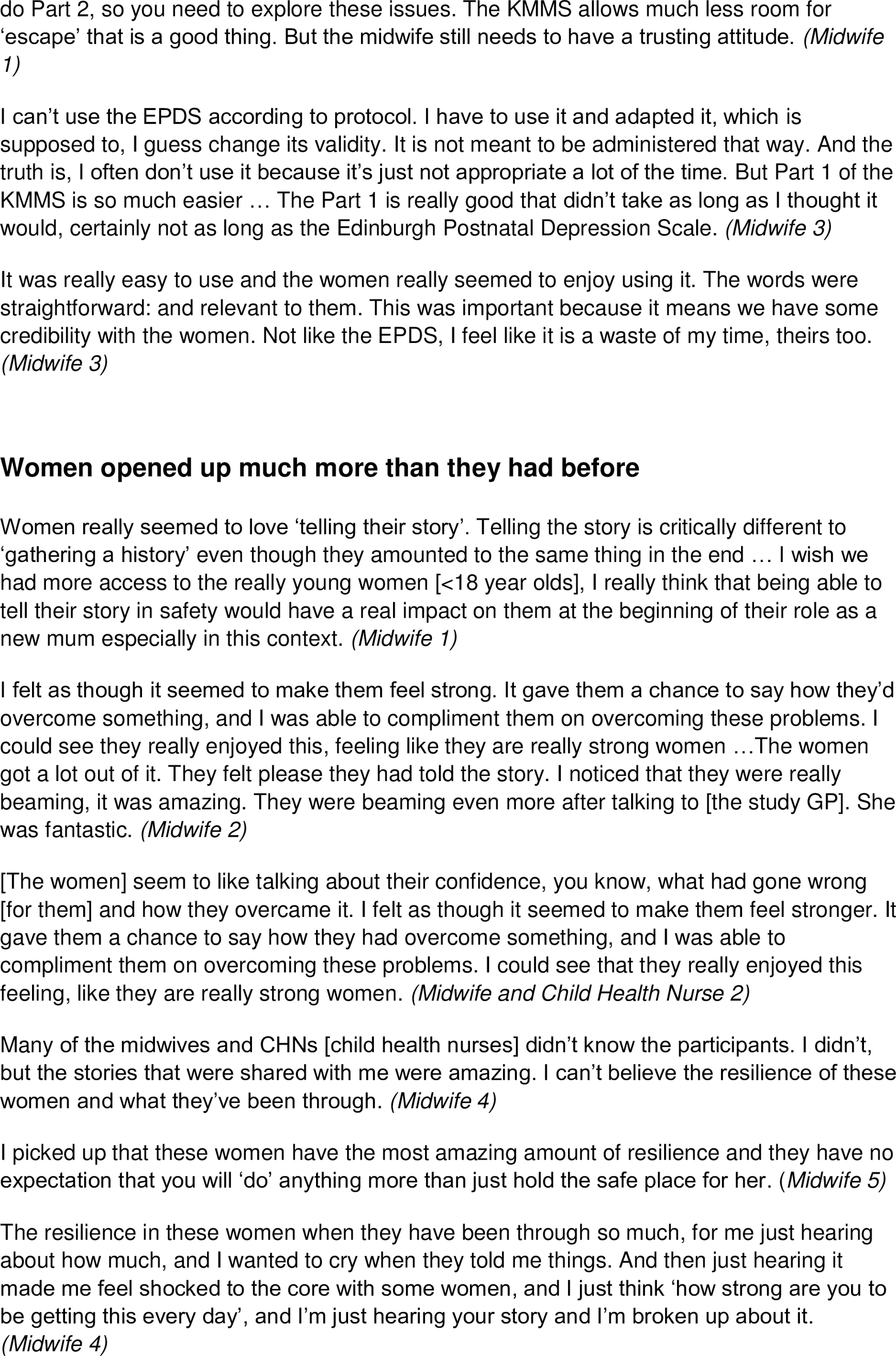

Supplement: S1 File — Kimberley Mum’s Mood Scale (KMMS) (Fig A). Validating the Kimberley Mum’s Mood Scale (KMMS) GP Data Collection Form (Fig B) Validating the Kimberley Mum’s Mood Scale (KMMS) Participant Feedback Form (Fig C). KMMS Study Personnel Online Questionnaire (Fig D). Further quotes illustrating the acceptability of the KMMS (Fig E). (ZIP) [file pone.0168969.s001.zip › S1 Appendix Fig E - 3.tif]

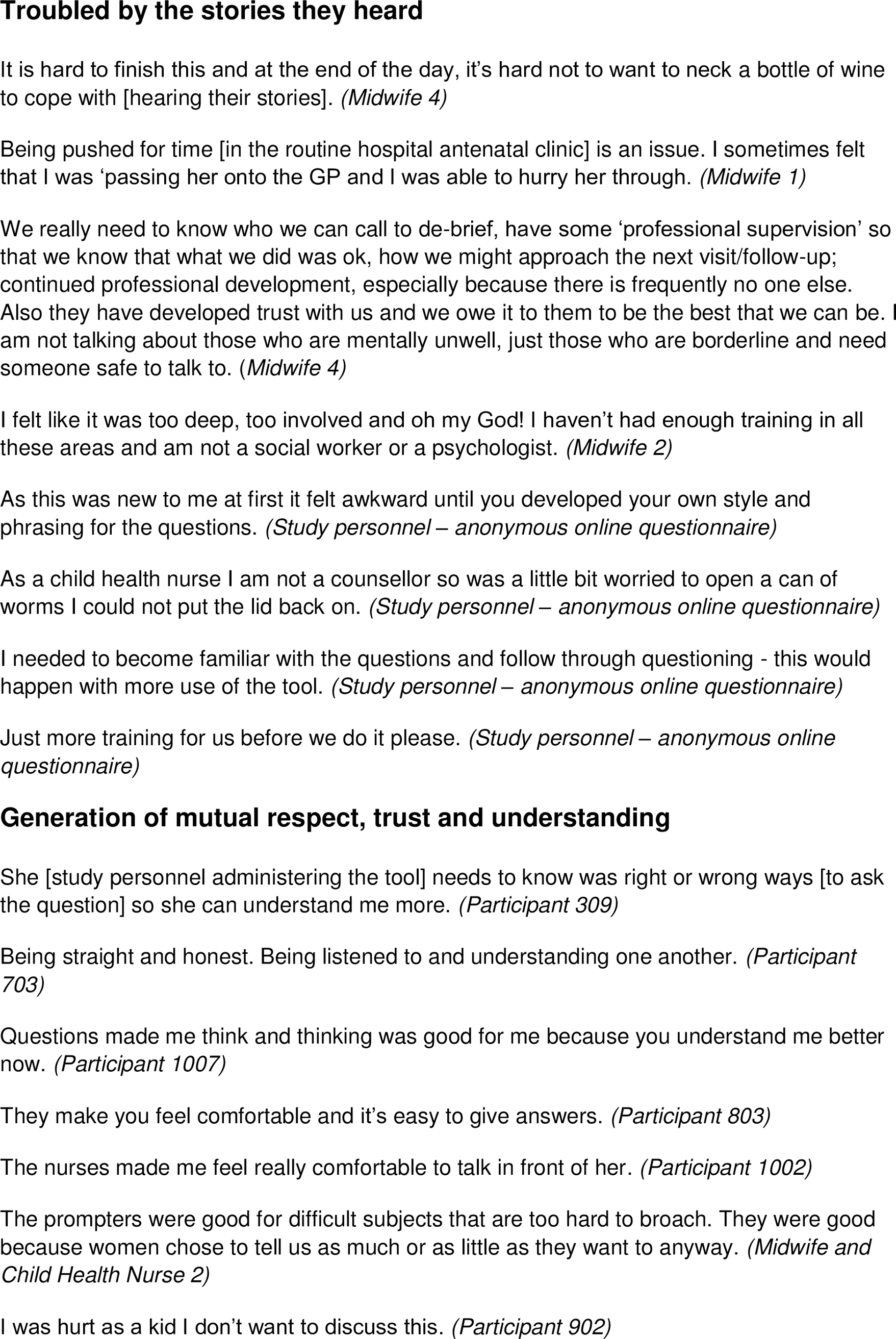

Supplement: S1 File — Kimberley Mum’s Mood Scale (KMMS) (Fig A). Validating the Kimberley Mum’s Mood Scale (KMMS) GP Data Collection Form (Fig B) Validating the Kimberley Mum’s Mood Scale (KMMS) Participant Feedback Form (Fig C). KMMS Study Personnel Online Questionnaire (Fig D). Further quotes illustrating the acceptability of the KMMS (Fig E). (ZIP) [file pone.0168969.s001.zip › S1 Appendix Fig E - 4.tif]
